# Diverse Horizontally Transferred Cellulose Biosynthesis Gene Clusters in *Escherichia coli* Strains

**DOI:** 10.3390/microorganisms14091932

**Published:** 2026-09-01

**Authors:** Seyedmohammad Hosseinpourlamardi, Shaiqa Labiba, Li Li, Ali Dadvar, Manita Guragain, Ute Römling

**Affiliations:** 1 Department of Microbiology, Tumor and Cell Biology, Biomedicum, Karolinska Institutet, 17177 Stockholm, Sweden; s.hosseinpourlamardi@gmail.com (S.H.); shaiqalabiba@gmail.com (S.L.); li.li@ki.se (L.L.); ali.dadvar@ki.se (A.D.); 2 Characterization and Interventions for Foodborne Pathogens, Agricultural Research Service, United States Department of Agriculture, Wyndmoor, PA 19038, USA; manita.guragain@usda.gov

**Keywords:** cellulose biosynthesis, *Escherichia coli*, cyclic di-GMP signaling, plasmid

## Abstract

The phosphoethanolamine-modified exopolysaccharide cellulose synthesized by the type IIa cellulose biosynthesis nanomachine is a major extracellular matrix component of *Escherichia coli*. Here, we aim to investigate whether homologs of *bcsABC* genes of the core genome EcType 1 *bcs* cellulose biosynthesis gene cluster, which are diminished or abolished in high-virulence isolates, and entire clusters are mobilized on plasmids and occasionally manifested on *E. coli* chromosomes at alternative locations. While EcType BcsA2 and BcsA3 cellulose synthases are restricted to the genus *Escherichia*, the EcType BcsA4 cellulose synthase and associated *bcs* gene products are highly similar to *Klebsiella pneumoniae* counterparts. Cyclic di-GMP turnover proteins not previously recognized to post-translationally regulate cellulose biosynthesis on the chromosomal level are frequently co-localized with novel *bcs* genes. Thermotolerant meat-derived *E. coli* 730V1 uniquely harbors an EcType 4 *bcs* gene cluster of type Ib with a type IIb *bcsG* gene on a plasmid. Chemical, phenotypic and genetic evidence showed cellulose biosynthesis, cellulose-dependent cell aggregation and activation of biosynthesis by the second messenger cyclic di-GMP, an allosteric activator of the cellulose synthase. With gene duplication, horizontal gene transfer and recombination to contribute to the multiplication and diversification of *bcs* gene clusters in a number of different bacterial species, the extent and ecological role(s) of the multiplication, transfer and replacement of cellulose biosynthesis gene clusters, as well as in species other than *E. coli*, still need to be unraveled. Our data indicate, however, that although diversification and multiplication of *bcs* clusters is ongoing in the species *E. coli*, these events become rarely manifested in the population.

## 1. Introduction

Poly 1,4-beta-D-glucan macrofibrills are an exopolysaccharide structure synthesized by bacteria, oocytes, animals and plants [1,2,3,4]. With the linear glucan chains tightly assembled in parallel into micro- and macrofibrills by weak, but numerous, hydrogen bonds, the chemically stable cellulose I macromolecule is formed in bacteria exemplified by *Komagataeibacter* spp. as major model organisms. Widespread in diverse branches of the bacterial domain of life, cellulose is a component of the extracellular matrix of biofilms; multicellular, predominantly sessile, assemblies of autonomous microbial cells. Biofilm formation can display as cell aggregates; microbial communities on abiotic and biotic surfaces; or pellicle formation at the air–liquid interface among other biofilm modes of growth [5]. Thereby, the catalytic core unit of cellulose biosynthesis, the cellulose synthase BcsA (also known as CelA) is embedded in different genomic contexts with different accessory elements [5]. For example, in Gram-negative bacteria, the *bcsA* cellulose synthase gene colocalizes with *bcsB*, encoding a periplasmic protein that interacts with a C-terminal transmembrane helix with BcsA and is essential for the catalytic activity of the cellulose synthase complex. Further, *bcsC* codes for the outer membrane pore to release the glucan chain from the periplasm into the extracellular space. BcsZ coding for a glycoside hydrolase GH family 8 glucanase (which can be replaced by a GH family 5 glucanase in other genetic context [6]) is the fourth gene of the core unit required for cellulose biosynthesis with a still not fully characterized role in biosynthesis and ecology [7,8,9,10].

Current experimentally verified cellulose synthases can be categorized into at least four different types associated with different accessory genes. Type I comprises the cellulose biosynthesis gene cluster present in the fruit-rotting alpha-proteobacterium *Komagataeibacter xylinus* (Ia), a model organism which has been shown to produce high amounts of unmodified poly-1,4 beta-glucan chains, the beta-proteobacterium *Burkholderia phymatum* (Ib) and the gamma-proteobacterium *Dickeya dadantii* (Ic) cellulose gene clusters as representatives. Type II is represented by the cellulose biosynthesis gene clusters of *Salmonella enterica* serovar Typhimurium (IIa), *Pseudomonas putida* KT2440 (IIb), *Burkholderia mallei* ATCC 23344 (IIc) and *Chromobacterium violaceum* ATCC 12472 (IId). Type III cellulose biosynthesis gene clusters are represented by *Agrobacterium fabrum* C58 (IIIa), *Methylobacterium extorquens* PA1 (IIIb), *Azospirillum lipoferum* 4B (IIIc) and *Acidiphilium cryptum* JF-5 (IIId). Type IV includes *Nostoc punctiforme* PCC 73102 (IVa) and *Nostoc* sp. PCC 7120 (IVb). Type I, II and III cellulose biosynthesis gene clusters are present in Gram-negative bacteria and are characterized by the presence of BcsB. The tight functional coupling is indicated by the occasional observation of BcsAB fusion proteins.

The bacterial cellulose macromolecule can be covalently modified by accessory gene products [11,12]. Thereby, type II cellulose biosynthesis gene clusters are characterized by the *bcsEFG* accessory genes. Their gene products are required for optimal expression/integration of the cellulose synthase into the cytoplasmic membrane, synthesis of the beta-1,4-glucan chain and the covalent modification of the nascent glucan chain with phosphoethanolamine at the C6 of the glucose [11,13]. Acetylation is another covalent modification of the 1,4-beta-glucan chain performed by gene products of the *wssFGHIJ* operon [12]. Despite possessing the same substrate specificity leading to the same product output, cellulose synthases and other gene products of the cellulose biosynthesis gene cluster can display a high sequence diversity [14]. On the other hand, recently, the first enzymes that synthesize mixed (1-3; 1-4) β-glucans have been identified in bacteria [15,16]. Those enzymes are highly homologous to cellulose synthases.

The type IIa cellulose biosynthesis gene cluster is highly conserved between *Escherichia coli* and *Salmonella typhimurium* with respect to gene conservation and chromosomal location [17]. In these two species, cellulose, often co-synthesized with the equally conserved amyloid curli fimbriae, constitutes one of the two major components of the extracellular matrix of the rdar (red, dry and rough colony morphotype) biofilm. In this agar-grown biofilm model, coloration and colony morphology, upon uptake of the diazo dye Congo red, are associated with the production of the extracellular matrix components [18]. While cellulose biosynthesis results in a pdar (pink, dry and rough) colony biofilm, curli expression yields a bdar (brown, dry and rough) colony biofilm. A saw (smooth and white) colony morphotype is obtained without expression of cellulose and curli extracellular matrix components.

Although rdar biofilm has initially been observed as a colony morphotype on agar plates, rdar biofilm and cellulose biosynthesis contribute to environmental persistence and tolerance against stress factors. Thereby the degree of rdar biofilm expression, in particular the biosynthesis of cellulose, balance environmental survival against acute virulence in the *Salmonella*-susceptible mouse model, including growth within macrophages [7,19,20]. Thereby, temperature-independent semi-constitutive rdar morphotype expression such as in *S. typhimurium* MAE52 possess decreased virulence in the mouse model allowing systemic infection [20]. Indeed, invasive, more virulent pathovars of *E. coli* and *S. typhimurium*, such as *Shigella* spp., Enteroinvasive *E. coli*, other *E. coli* sequence types (ST) and invasive *S. typhimurium* ST313, have highly reduced or entirely lost the ability to produce full rdar biofilms, including the ability to synthesize cellulose [21,22,23]. Surprisingly though, in the case of *Shigella*, some of these pseudogenes have regained functionality under in vivo-like conditions [24].

On the other hand, certain bacterial species, such as *Komagataeinbacter* spp. which produce high amounts of cellulose, possess more than one cellulose biosynthesis gene cluster [25]. This is especially surprising in the case of *Proteus* spp. which have not been reported to synthesize cellulose [14]. Of note, *Proteus* species possess only one copy of a catalytically competent GGDEF diguanylate cyclase producing the ubiquitous second messenger cyclic di-GMP which is hypothesized to post-translationally stimulate cellulose biosynthesis via the PilZ domain located at the C-terminal end, as in most bacterial cellulose synthases [14].

However, the role of cellulose biosynthesis in *E. coli* is more complex than previously anticipated. In this work, we report alternative horizontally transferred cellulose biosynthesis gene clusters coding for distinct cellulose synthases which contain a variable number of essential and accessory gene products in *E. coli* strains. Thereby, alternative cellulose biosynthesis gene clusters EcType 2 and 3, which conventionally consist of the core components *bcsABC*, were found to be plasmid- or chromosomally encoded, while an EcType 4 *bcs* gene cluster introduced novel *bcs* cluster-associated genes such as *bcsD* into *E. coli*. The plasmid-encoded *bcs4* gene cluster of the meat-derived thermotolerant *E. coli* strain 730V1 is functional and can be activated by the second messenger cyclic di-GMP. Further experimentation indicated synthesis of cellulose from the plasmid-encoded EcType 4 *bcs* gene cluster under distinct nutrient conditions and oxygen pressure. These observations indicate that genes that code for exopolysaccharide extracellular matrix components can be highly mobile even in human-associated bacteria. The evolutionary forces that drive the dissemination and diversification of cellulose biosynthesis genes in *E. coli*, including the thermotolerant meat-derived strain *E. coli* 730V1, and other bacterial species including plant symbionts still need to be unraveled.

## 2. Materials and Methods

### 2.1. Bacterial Strains and Growth Conditions

The following strains were used in this work: high temperature-tolerant *E. coli* 730V1 isolated from beef [26] which harbors a *bcs* EcType 4 cellulose biosynthesis gene cluster on its large plasmid pUnnamed 1 (pU1) and *E. coli* 730-2 (*stx*^+^) isolated from the same sample. Reference strains were *S. typhimurium* UMR1 (ATCC 14028 Nal^r^ rdar_28_), its temperature-independent variant MAE52 (UMR1 P*csgD1*) [27] and the respective only-cellulose mutant MAE97 (MAE52 Δ*csgBA102*; pdar_28/37_), only-curli mutant MAE171 (MAE52 Δ*bcsA102*; bdar_28/37_), and curli- and cellulose-negative mutant MAE777 (Δ*bcsA102* Δ*csgBA::Km*, saw_28/37_). Microaerophilic conditions with high CO_2_ (O_2_ = 5%, CO_2_ = 10%, N_2_ = 85%) were created with the CampyGen 2.5L gas pak (Thermo Scientific, Waltham, MA, USA) inside an airtight container. Tetracycline was added at 20 µg/mL, kanamycin at 50 µg/mL, nalidixic acid at 50 µg/mL and L-arabinose at 0.1% (*w*/*v*), if required.

### 2.2. Selection of an E. coli 730V1 Nalidixic Acid-Resistant Variant

*E. coli* 730V1 cells were incubated overnight in LB medium at 37 °C and subsequently centrifuged at 7000 rpm for 1 min. A 200 µL cell suspension adjusted to OD_600_ = 10 was spread on an LB agar selection plate containing 50 μg/mL nalidixic acid. After incubation for three days, two of the colonies that grew on the selection plate were streaked twice to obtain single colonies and subsequently stored at −80 °C as a glycerol stock. The resulting nalidixic acid-resistant strains were then investigated for rdar morphotype expression.

### 2.3. Plasmid Conjugation by Triparental Mating

First, 500 µL of overnight cultures of the donor strain *E. coli* containing pWJB9 (pLAFR3 containing the diguanylate cyclase AdrA under the pBAD promoter [28]) or the pLAFR3 vector control, *E. coli* containing the helper plasmid pRK2013, and the recipient strain *E. coli* 730V1 Nal^r^ were mixed and subsequently centrifuged at 13,000 rpm for 1 min. The pellet was resuspended and 50 µL spotted onto an LB plate without antibiotics for overnight incubation at 37 °C. Cells were subsequently resuspended in 10 mM MgSO_4_, washed once and spread onto an LB agar plate containing nalidixic acid (50 μg/mL) and tetracycline (10 μg/mL). The plate was incubated for three days at 37 °C to recover plasmid-containing strain *E. coli* 730V1 Nal^r^ colonies. At least two of these colonies were streaked onto LB agar plates containing the antibiotics at least twice to obtain purified single colonies.

### 2.4. Deletion of the bcsA4 Gene of E. coli 730V1

Deletion of the *bcsA4* cellulose synthase gene of *E. coli* 730V1 was performed by one step gene inactivation [29]. Vector pKD46/pKD88 encoding the λ red recombinase was electroporated into *E. coli* 730V1. The chloramphenicol and kanamycin antibiotic resistance cassettes of pKD3/pKD4, respectively, were amplified by PCR with primers containing 39 bp nucleotide sequences at the 5′ end homologous to the 5′ and 3′ ends of the target gene. The PCR product was subsequently transformed into *E. coli* 730V1 by electroporation, recombinants selected and tested for gene replacement by PCR using primers outside of the recombination region. Primers and plasmids are displayed in Table 1.

### 2.5. Bioinformatic Analyses

Complete plasmids from microbes (NCBI microbial database and the curated NCBI database at https://ccb-microbe.cs.uni-saarland.de/plsdb2025 (last access on 14 August 2026)) and the NCBI database of complete and draft genomes were searched for homologs of the core genome cellulose synthase BcsA1 (P37653) of *E. coli* MG1655 using default parameters. All non-redundant homologs on plasmids and distantly related homologs on *E. coli* chromosomes with an apparent query coverage of >47% and 40% were retrieved [30]. Truncated proteins were removed from the analysis. Proteins were aligned using ClustalX 2.1 [31] or Clustal Omega (https://www.ebi.ac.uk/jdispatcher/msa/clustalo?stype=protein (access on 14 August 2026) [32]) with the alignment to be manually curated in GeneDoc 1.0 [33]. Identity and similarity values for proteins were calculated using the .fasta version of the alignments with ‘Ident and Sim’ with similar amino acids classified according to GAVLI, FYW, CM, ST, KRH, DENQ, and P at https://www.bioinformatics.org/sms2/ident_sim.html (access on 14 August 2026) [34]. For phylogenetic analysis, protein sequences were trimmed to contain the homologous regions common to all proteins. Protein alignments were displayed by ESPript 3.0 [35]. Maximum-Likelihood pylogenetic trees were constructed with MEGA 11.0 [36] or IQ-TREE 2 [37]. The BcsA cellulose synthases were subsequently categorized according to the degree of homology in three additional EcTypes, BcsA2, BcsA3 and BcsA4, and, if required, their origin as cellulose synthases of *E. coli* was verified by the phylogenetic assessment of conserved proteins informative on the species level such as GyrA. BcsA2 (WP_074014783.1) from strain *E. coli* S56 on plasmid pA, BcsA3 (HDP8224287.1) from strain *E. coli* ST-2070 (identical to the one from *E. coli* 21-X O621GT02-EC), and BcsA4 (WP_004186066.1) from strain *E. coli* 730V1 on plasmid pU1 were chosen as representative proteins. Reference BcsA cellulose synthases include type I–IV cellulose synthases [5], cellulose synthases most closely related to *E. coli* BcsA2-BcsA4 cellulose synthases, and additional more distantly related cellulose synthases from the database as published previously [38]. A similar strategy to retrieve homologous proteins was applied for all alternative cellulose biosynthesis proteins identified as encoded by *E. coli* genomes besides those encoded by the core cellulose biosynthesis gene cluster.

The gene neighborhood of the BcsA cellulose synthase genes (of 62 *E. coli* and 80 non-*E. coli* non-redundant BcsA proteins) was assessed manually for each non-redundantly retrieved BcsA cellulose synthase by evaluating the annotated chromosomes and plasmids. After identification of the cellulose biosynthesis gene cluster, the cluster +/− four genes up- and down-stream was downloaded. Cellulose biosynthesis gene clusters were comparatively visualized with Easyfig [39] with the most conserved BcsA cellulose synthase as reference.

Proteins (including cellulose biosynthesis operon gene products and GGDEF/EAL domain proteins) were aligned with ClustalX 2.1 and manually curated in Genedoc [31]. If required, the analysis was restricted to a single domain, as in the case of GGDEF and EAL domain proteins. Multiple alignments with reference proteins were displayed with ESPript 3.0 [35]. A Maximum-Likelihood phylogenetic tree was constructed in MEGA 7.0 or MEGA 11.0 using 1000 bootstrap iterations [36].

Transmembrane helices were evaluated with DeepTMHMM 1.0, TMHMM 2.0 [40] and TOPCONS (https://single.topcons.net/) [41]. Transmembrane helices and domain borders of BcsA cellulose synthases were evaluated against the crystal structure of the *Cereibacter* (previously *Rhodobacter*) *sphaeroides* cellulose synthase (PDB: 4HG6). Conserved signature amino acids for the cellulose synthase were taken from the literature [42,43].

Domain structures were displayed with Illustrator for Biological Sequences IBS 2.0 [44]. Protter (https://protter.ethz.ch/start/) [45] was used to visualize the amino acid sequences within the domain structures.

FimH types, serotypes and plasmids’ origin of replication were identified using servers provided by the Center of Genomic Epidemiology, FimTyper 1.0, SerotypeFinder 2.0 and PlasmidFinder 2.1, respectively [46]. All BcsA- and PgaC-bearing plasmids were retrieved from the PLSDB database at https://ccb-microbe.cs.uni-saarland.de/plsdb2025/ (last access on 22 November 2024) [47]. Representative plasmids were displayed by Proksee (https://proksee.ca/; [48]) reannotated with Prokka [49].

### 2.6. Box Plot Calculation with R

Mean and median plasmid sizes were calculated, and a box plot showing the first and third quartiles with borders was drawn using R studio version 4.4.

### 2.7. Assessment of Cell Aggregation, Biofilm Formation, Cellulose Expression and Colony Morphotype

Bacteria were recovered from −80 °C stocks by streaking onto an LB agar plate. After overnight growth, colonies were resuspended in LB without NaCl (LB-NS) medium and adjusted to OD = 5. Subsequently, 5 µL of cell suspension was spotted on LB-NS agar plate supplemented with Congo red (CR) (CR 40 µg/mL and Coomassie Brilliant Blue 20 µg/mL) or Calcofluor white (fluorescence brightener 28, 50 µg/mL) and allowed to dry [50]. Alternatively, Tryptone (1% tryptone) medium was used in agar plates. Colony morphology was assessed visually or by observing the cells exposed to a 365 nm UV light source after 24, 48 and 72 h of growth at 28 °C, 37 °C or 42 °C and documented by photographing. To induce the production of the AdrA diguanylate cyclase under the control of the pBAD promoter, 0.1% L-arabinose was added to Congo red and Calcofluor white agar plates [28].

Biofilm formation and cell aggregation in liquid culture were assessed after either aerobic or microaerophilic growth (achieved either by differential filling of the flask and shaking or by use of the CamyGen 2.5 L gas pak, which also provides 10% CO_2_) in LB-NS and M9 medium supplemented with 0.2 or 0.4% glucose [51]. Culture flasks were photographed at the end of the incubation period to document visible aggregation, and bacterial aggregates were subsequently observed under a fluorescence microscope (Nikon Eclipse 80i (Nikon, Tokyo, Japan)) using an excitation wavelength of 340–380 nm and observing emission at a wavelength of 435–485 nm after staining with fluorescent brightener 28 (Calcofluor white).

### 2.8. Extraction of Cellulose from Bacterial Cultures

Cellulose has been extracted by two different approaches. Crystalline cellulose was extracted by the Updegraff reagent (58% acetic acid, 19% nitric acid) [52] at 95 °C for 30 min, and the pellet was subsequently washed with double distilled H_2_O.

Extraction of crystalline and amorphous cellulose by alkaline treatment:. Cells grown in M9 minimum medium with 0.4% glucose under microaerophilic conditions were harvested after 24 h incubation, resuspended in 1% potassium hydroxide and incubated for 1 h at 95 °C under strong shaking. The sample was centrifuged at 13,300 rpm for 10 min, and the pellet was washed three times with water.

### 2.9. Analysis of Cellulase-Digested Products by Thin-Layer Chromatography

KOH-isolated cellulose from *E. coli* 730V1 was digested with 0.1 mg/mL cellulase from *Trichoderma viride* (Sigma-Aldrich, St. Louis, MO, USA) in 0.05 M sodium citrate pH 4.6 overnight. The reaction mixture was dried, dissolved in water and centrifuged at 13,000 *g* for 15 min. Then, 2 μL of the sample was applied on a 5 × 20 cm silica-based thin-layer chromatography plate (TLC plates, silica gel 60 F_254_, (Supelco, Bellefonte, PA, USA)) and 3 µL of a glucose and cellobiose solution (5 mg/mL) was used as standard. The solvent system consisted of n-butanol, acetic acid and water (2:1:1), respectively. After drying, the plate was immersed in the dipping solution orcinol (0.2 g/100 mL) in sulfuric acid (20 g/100 mL) and heated for 10 min at 100 °C for visualization of the carbohydrates [53].

## 3. Results

### 3.1. Escherichia coli Strains Can Possess Two BcsA Cellulose Synthases

Not only highly cellulose-producing bacterial species like *Komagataeibacter* spp. possess more than one cellulose biosynthesis gene cluster, even bacterial species/strains belonging to the families Morganellaceae and Enterobacteriaceae, such as *Proteus* spp. and *Klebsiella* spp., respectively, possess more than one cellulose biosynthesis gene cluster [5,14,17]. Commonly, isolates of the species *E. coli* are considered to encode one conserved type IIa cellulose biosynthesis gene cluster consisting of the two divergently transcribed operons *bcsQRABZC* and *bcsEFG* on the core genome [17,54]. *E. coli* strains, as well as the closely related *Salmonella* spp., have been observed, upon evolution, to lose the ability to synthesize cellulose [21,22]. However, upon investigating the conservation of cellulose synthases in *E. coli* by BLAST screening for homologous proteins, we noted in certain *E. coli* strains the occurrence of cellulose synthases only distantly related to the core genome BcsA1 cellulose synthase. We therefore set up to investigate the diversification and the prevalence of alternative cellulose biosynthesis gene clusters in *E. coli* in more detail.

**Figure 1:**
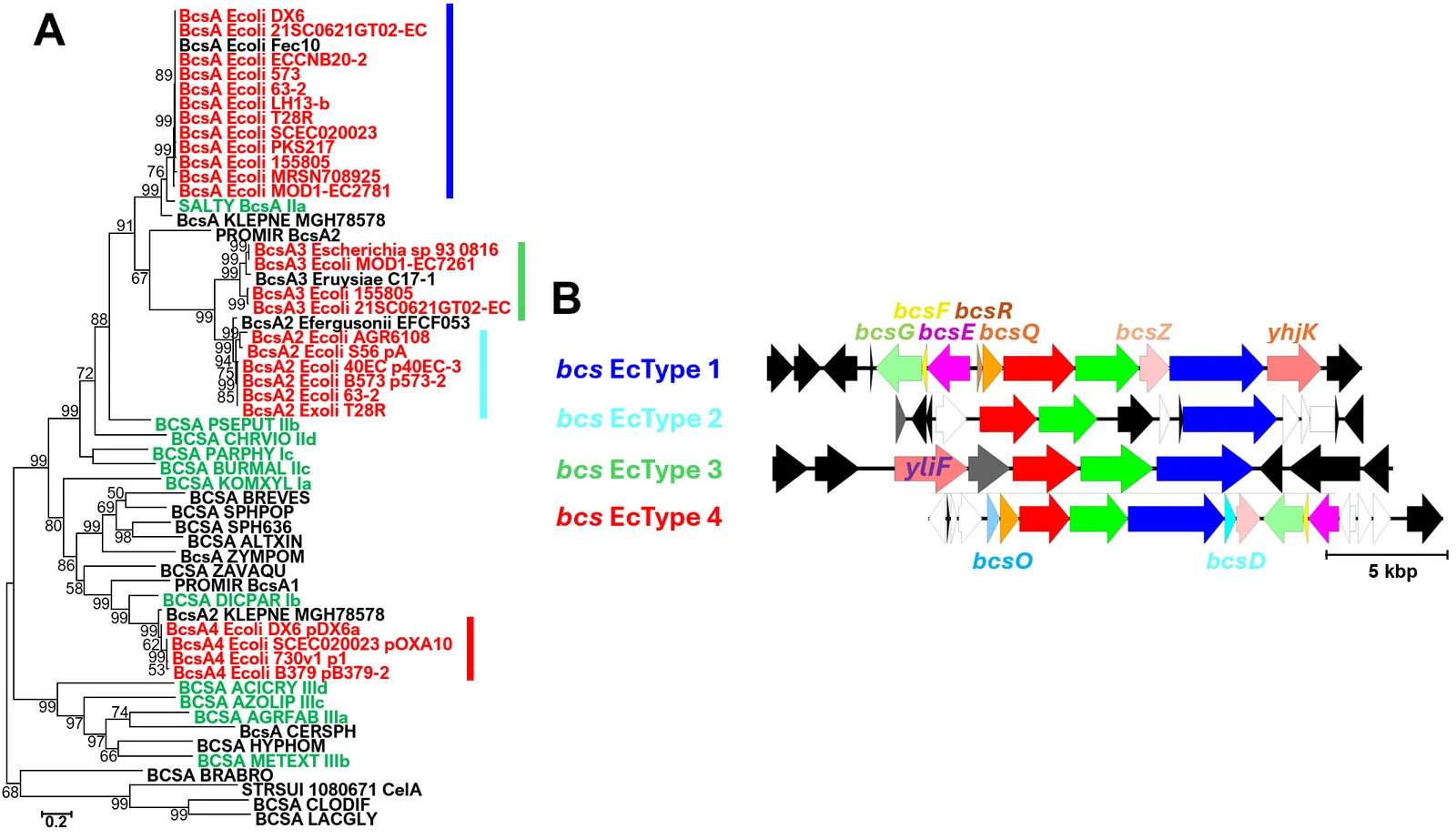
Four distinct cellulose synthase and cellulose biosynthesis gene clusters exist in *E. coli* strains. (**A**) Phylogenetic tree of representative *E. coli* cellulose synthases BcsA1-4 with reference BcsA proteins and genomic context of *bcsA1-4* genes. Selected BcsA cellulose synthases (see Supplementary Material and Figure S1A) were aligned with ClustalX 2.1 [31] and subsequently manually curated in GeneDoc. The phylogenetic relationship has been assessed using a Maximum-Likelihood (ML)-based phylogenetic tree with 1000 bootstraps in MEGA 11.0 [36]. The nucleotide sequences and annotations of the *bcs* gene clusters and their gene neighborhoods were extracted from the genomic sequences stored at NCBI and aligned and visualized with Easyfig [39]. *E. coli* and *Escherichia* BcsA sequences are shown in red and reference BcsA sequences [5] are shown in green. Size bar, substitutions per site. (**B**) Representative bacterial cellulose biosynthesis (*bcs*) gene clusters of the four EcTypes: EcType 1, core genome *bcs* gene cluster encoding BcsA1 cellulose synthase; EcType 2-4, accessory *bcs* gene clusters encoding BcsA2-4 cellulose synthases. Designation of gene identity, see Figure S1 (red arrow, *bcsA*; green arrow, *bcsB* and dark blue arrow, *bcsC*). White arrow, mobile element gene.

A systematic analysis of the presence of distantly related BcsA cellulose synthases outside of the core genome type IIa (EcType 1) cellulose biosynthesis gene cluster in *E. coli*, both on the core genome and on plasmids, uncovered three additional groups of cellulose synthases termed BcsA2-BcsA4, each with distinct similarity to the core genome cellulose synthase BcsA1 and to each other (Figure 1A and Figure S1A,B; Table S1).

BcsA2 members show approximately 43% identity and 57% similarity, BcsA3 members 41%/55% and BcsA4 members 35%/50% as compared to the core genome cellulose synthase BcsA1 (Figure 1A and Figure S1A,B). The percent amino acid sequence identity/similarity between the different types is highest at 73.5%/83.9% between BcsA2 and BcsA3, and approximately 40%/59% between BcsA2/BcsA3 and BcsA4. Within one type, the percent amino acid identity is >90%. BcsA2 and BcsA3 can be discriminated by characteristic amino acid signatures present in all members (Figure S1B). For example, the ADPIL motif in BcsA2 has been replaced by the ENKEE motif and the HTGDEE motif replaced by DDSNGK/E. Of note, most amino acid substitutions were observed in the PilZ domain. We noted that BcsA2 and BcsA3 cellulose synthase proteins are not found outside of the *Escherichia* genus, but were occasionally observed in other *Escherichia* species (BLAST analysis, Figure 1A and Figure S1A). However, the BcsA4 EcType 4 cellulose synthase, most distantly related to BcsA1, is most closely related to one of two cellulose synthases of *Klebsiella pneumoniae* MGH 7843 and clusters with the type Ib cellulose synthase of *D. dadantii* (Figure S1). BcsA2 to BcsA4 cellulose synthases, as the vastly investigated cellulose synthase of *Cereibacter sphaeroides* [55], lack the extended approx. 150-amino-acid-long N-terminal end of *E. coli* BcsA1 but maintain the eight core transmembrane helices flanking the catalytic cytoplasmic domain. Further more, the signature amino acids indicative for processive family 2 glycosyltransferase (GT2; www.cazy.org) catalytic activity, cellulose synthase activity and the C-terminal PilZ domain with the RxxxR and DxGxS binding motifs required for cyclic di-GMP binding are present (Figure 1A and Figure S1A,B). BcsA2-BcsA4, however, lack the extended C-terminus of approx. 30 amino acids. As concluded from these analyses, BcsA2 to BcsA4 cellulose synthases are predicted to be functional.

### 3.2. BcsA2, BcsA3 and BcsA4 Are Found in Different Genetic Contexts

**Figure 2:**
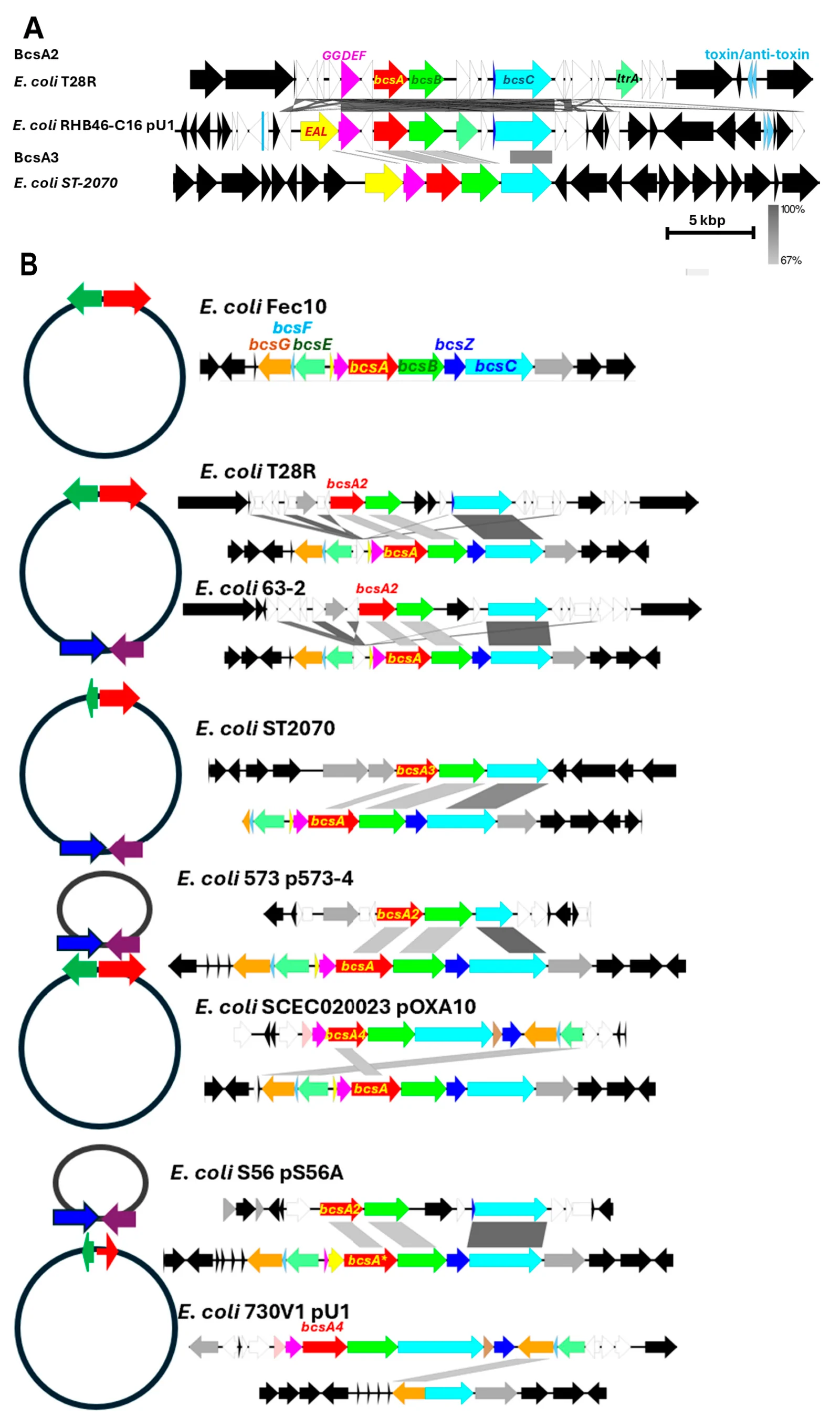
Bacterial cellulose biosynthesis gene clusters in the genomic context in *E. coli* strains. (**A**) Chromosomal and plasmid BcsA2 and BcsA3 cellulose biosynthesis gene clusters. White arrow, mobile element gene. (**B**) Occurrence of *bcsA2-4* (EcType 2-4) cellulose synthase gene clusters in different contexts in *E. coli*: left: conventional on chromosome (green arrow *bcsRQABZC*; red arrow, *bcsEFG* EcType1 operons); conventional + unconventional (blue and violet arrows) on chromosome; conventional on chromosome and unconventional on plasmid; conventional deleted. Reference strain for a functional EcType 1 *bcs* gene cluster is *E. coli* Fec10, a modern commensal human ST10 isolate [57], clonal to *E. coli* K-12 MG1655. The nucleotide sequences and annotations of the *bcs* gene clusters and their gene neighborhoods were extracted from the genomic sequences stored at NCBI, aligned and visualized with Easyfig [39]. Gray arrow, cyclic di-GMP turnover protein gene; white arrow, mobile element gene.

The three cellulose synthase EcTypes BcsA2, BcsA3 and BcsA4 genes are integrated in different genetic contexts (Figure 1A and Figure S2, Table S1). We observed that the BcsA2 cellulose synthase gene is, in the majority of cases, found embedded in a *bcsABC* gene cluster often located on plasmids >90 kbp in size with a p0111 origin of replication, indicating phage origin of the plasmid [56], but it can also be found, exceptionally, on the chromosome (for example, *E. coli* strain T28R (Figure 2)). Those *bcs*-bearing plasmids are on average larger than *E. coli* plasmids without *bcsA* (Figure 1A, Figures S2 and S3).

As far as can be concluded from the available sequenced genomes and the genetic context on contigs, *bcsA3* genes are embedded in a *bcsABC* gene cluster located on the chromosome. Located within an extended *bcs* gene cluster, BcsA4 cellulose synthase encoding genes are encoded on large plasmids in the context of *bcsOQABCDZ bcsEFG* gene clusters. The entire BcsA4 gene cluster is highly similar to a chromosomally encoded gene cluster from *Klebsiella* spp. (Figure S1). With the cellulose synthases being highly similar to type Ib, the EcType 4 cellulose biosynthesis gene cluster is a hybrid of the type Ib and type II cellulose biosynthesis gene cluster.

We subsequently investigated whether gene products associated with the BcsA cellulose synthases have a similar phylogenetic clustering which would indicate a common horizontal gene transfer or whether a mosaic structure of gene acquisition needs to be considered. To this end, we aligned and subsequently constructed phylogenetic trees of all the cellulose biosynthesis proteins that had been identified in the BcsA2-BcsA4 cellulose synthase gene clusters in the context of reference proteins (Figure S4). We found that BcsB, required for the catalytic activity of the cellulose synthase (and is even occasionally found in E. coli as a fusion protein with BcsA (Figure S1A)), and the outer membrane pore BcsC cluster concordantly as BcsA (Figure S4). Also the phylogenetic context of the cellulase BcsZ is congruent with the phylogeny of BcsA (Figure S4). BcsE and BcsG members of the core genome cellulose biosynthesis gene cluster are present in the context of the type Ib (EcType 4) BcsA4 cellulose biosynthesis gene clusters (Figure S4). *BcsE4* encoding the GIL-type cytoplasmic c-di-GMP receptor required for optimal cellulose biosynthesis [58] has, however, consistently evolved as a pseudogene. BcsG4 members, required for the stabilization of the cellulose synthase and covalent modification of the glucan chain [11,13], on the other hand, built up their own cluster.

The accessory protein BcsD is located in the BcsA4-like gene cluster, consistent with being a component of the type Ib cluster [5,59]. BcsD enhances the crystallinity of the cellulose macromolecule by interaction with the cellulose synthase complex and the nascent glucan chain (Figure S4; [60]). The accessory protein BcsO is conventionally also found in Type Ib *bcs* gene clusters [5]. Indeed, BcsO is infrequently found in cellulose biosynthesis gene clusters. Proline-rich BcsO interacts in the cytoplasm with BcsD in *D. dadantii* Ech703 and other plant-associated or environmental gamma-proteobacterial species (Figure S4, [60]). We identified the closest homologues of BcsO by BLAST search. The phylogenetic tree of the evolutionary relationship showed that BcsO is highly similar to *K. pneumoniae* BcsO present in the second cellulose biosynthesis operon (Figure S4).

### 3.3. The wss Genes Form a Novel Type of Cellulose Biosynthesis Operon Producing Acetylated Cellulose

The phylogenetic trees for the BcsA cellulose synthase and the periplasmic BcsB protein included several reference sequences (Figure 1A and Figure S1A,C): 1. the representative cellulose synthases of type I to IV, cellulose biosynthesis gene clusters and respective subtypes, and 2. confirmed and 3. to-be-confirmed BcsA cellulose synthases as investigated in the literature, but not classified to any type. While cellulose synthases of different types predominantly form phylogenetically coherent clusters, exceptions such as the EcType 4 gene cluster exist (Figure 1A and Figure S1A). Thus the BcsA4 cellulose gene cluster is predicted to synthesize phosphoethanolamine-modified cellulose despite being a type Ib core gene cluster. The Wss cellulose biosynthesis gene cluster is characterized by accessory genes involved in the partial acetylation of various carbon positions at the glucose moieties [12]. Its cellulose synthases form a phylogenetically coherent protein cluster. Thus, we propose that Wss-type cellulose biosynthesis gene clusters constitute a new type Id cluster (Figure S1A). Also, cellulose synthases more distantly related to type Ia, such as those present in the alpha-proteobacterium *Zymomonas mobilis* [38,61] and *Brevundimonas vesicularis* (Figure S1A), are suggested to be categorized into the new type Ie. We also cannot currently exclude that some of the proposed cellulose-like synthases such as CelA of Streptococcus spp. synthesize alternative exopolysaccharides and are, for example, mixed 1-3, 1-4 beta-glucan synthases (Figure S1C).

### 3.4. Horizontal Acquisition of a Second Cellulose Biosynthesis Operon Results in Different Outcomes

In contrast to the gene cluster for poly-N-acetyl-glucoseamine biosynthesis, the chromosomal location of the conventional *bcs* EcType 1 cellulose biosynthesis gene cluster is conserved [50]. We therefore wondered about the fate of the core genome *bcs* EcType 1 gene cluster upon acquisition of a second cellulose biosynthesis operon on the chromosome. Investigation of the reference strains harboring an alternative cellulose biosynthesis operon showed that various constellations can occur (Figure 1B, Figure 2 and Figure S2).

The newly introduced unconventional *bcs* gene cluster can be found either on a large plasmid or on the chromosome. Thereby, the location of the second *bcs* gene cluster on the chromosome is distinct from the *bcs* EcType 1 gene cluster. Independently of the chromosomal or plasmid location of the horizontally transferred gene cluster, the conventional *bcs* EcType 1 gene cluster is either present or has been deleted. Consequently, the biological impact of two cellulose biosynthesis gene clusters and the replacement of the core genome cellulose biosynthesis gene cluster needs to be unraveled in the appropriate isolates.

### 3.5. BcsA2-BcsA4 Are Located on Large E. coli Plasmids

Horizontally transferred *bcs* (EcType 2, EcType 3 and EcType 4) gene clusters can be located on large plasmids. Characterization of all *bcs*-bearing plasmids from the genus *Escherichia* (with a few from the *Shigella* pathovar and *Escherichia* species other than *E. coli*) showed that the majority of plasmids belonged to the incompatibility groups p0111 and IncFIB and had sizes between 39.17 kbp and 1310.597 kbp, with a mean size of 143.1 ± 112.4 kbp, which is larger than that of non-BcsA encoding plasmids (mean 74.8 ± 68.0 kbp) (Figure S3). The isolation source and origin of *E. coli* strains harboring *bcs*-bearing plasmids is diverse (Table S1), although the majority of strains were non-pathogenic and derived from environmental sources and birds collected in China and the United Kingdom. This is clearly a restricted isolation source and origin compared to all the *E. coli* strains harboring plasmids. Representative plasmids encoding *bcs* EcType 2, EcType 3 and EcType 4 cellulose biosynthesis gene clusters are shown in Figure S2.

### 3.6. GGDEF and EAL Domain Proteins Are Associated with Horizontally Transferred bcs Gene Clusters in E. coli

**Figure 3:**
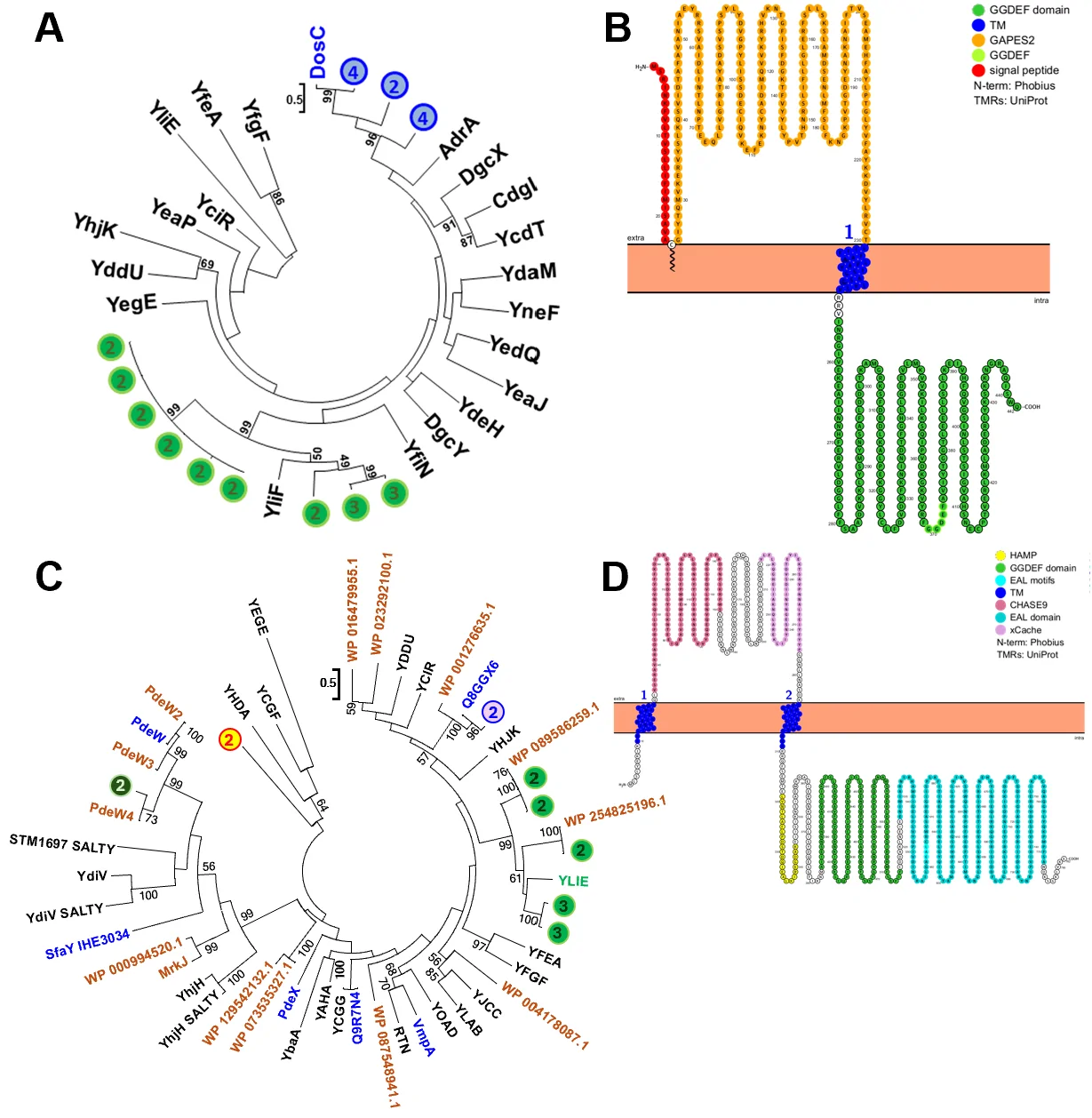
Phylogenetic tree to place GGDEF diguanylate cyclase and EAL phosphodiesterase domain proteins adjacent to *bcsA* EcType 2-4 cellulose biosynthesis gene clusters in the context of all chromosomally encoded cyclic di-GMP turnover proteins of *E. coli* MG1655 (identical with the modern ST10 strain *E. coli* Fec10 [57]). (**A**) Phylogenetic tree of *E. coli* GGDEF domains of GGDEF domain proteins found adjacent to BcsA2-4 cellulose biosynthesis gene cluster(s) in the context of the GGDEF domains from chromosomally encoded proteins. GGDEF domain proteins adjacent to BcsA2-4 cellulose biosynthesis operons are homologous to YliF and DosC over the entire length of the amino acid sequence (Figure S5). Numbers indicate the BcsA cellulose synthase class adjacent to the GGDEF domain protein. (**B**) Protter image of the chromosomally encoded GGDEF domain protein YliF as the GGDEF domain protein most frequently found to be encoded on BcsA encoding plasmids. (**C**) Phylogenetic tree of *E. coli* EAL domains of EAL domain proteins found adjacent to BcsA2-3 cellulose biosynthesis operons in the context of EAL domains from chromosomally encoded proteins. EAL domain proteins adjacent to BcsA2-3 cellulose biosynthesis gene clusters are homologous to the chromosomally encoded core genome YliE GGDEF-EAL domain protein over the entire length of the amino acid sequence (Figure S5). Other EAL domain proteins are homologous to PdeW and Q8GGX6 accessory EAL domain protein. In addition, an EAL domain protein with a novel N-terminal signaling protein has been identified. Numbers indicate the BcsA cellulose synthase EcType adjacent to the EAL domain protein. Green letters, YliE GGDEF-EAL domain protein most frequently found encoded adjacent to *bcs* gene clusters; black letters, EAL domain of core genome-encoded EAL domain protein of *E. coli* and *Salmonella typhimurium* (SALTY); blue letters, accessory EAL domain proteins experimentally investigated; brown letters, accessory EAL domain proteins not yet investigated. (**D**) Protter image of the chromosomally encoded CHASE9-xCache GGDEF-EAL domain protein YliE the EAL domain protein most frequently found to be encoded on BcsA encoding plasmids.

As a major stimulatory trigger, bacterial cellulose biosynthesis is post-translationally activated by the ubiquitous second messenger cyclic di-GMP [17,28,62]. Diguanylate cyclase activity is conducted by the GGDEF domain, while the EAL domain has phosphodiesterase activity [63,64]. We observed that GGDEF and EAL domain protein genes are commonly located immediately adjacent to chromosomally encoded *bcs* EcType 2 and EcType 3 gene clusters (Figure 1, Figure 3, Figures S1A and S2).

Thus, we hypothesize that the co-localization of these genes indicates connected functionality, namely that those cyclic di-GMP turnover proteins produce or hydrolyze this second messenger, which is required to stimulate cellulose biosynthesis. Subsequently, we investigated whether plasmids bearing *bcs* gene clusters also code for GGDEF and/or EAL domain proteins. Indeed, we identified GGDEF and EAL domain proteins encoded on *bcs*-bearing plasmids (Figure 3, Table S2). Subsequently, we were wondering about the identity of the GGDEF and EAL domain proteins and whether they have been previously identified to regulate cellulose biosynthesis in the native context. Of note, the chromosomally encoded GGDEF and EAL domain proteins adjacent to *bcs* EcType 2 and EcType 3 are homologous to plasmid-encoded GGDEF and EAL domain proteins. However, plasmid-encoded GGDEF and EAL domain proteins are more diverse.

The GGDEF protein YliF, one of the *E. coli* MG1655 core genome GGDEF proteins, has the TM-GAPES2-TM-GGDEF domain composition. YliF homologs located adjacent to chromosomal *bcs* EcType 2 and EcType 3 gene clusters are most frequently found on *bcs*-bearing plasmids, with the three major variants 39, 32 and 41% identical over the entire length of the protein compared to YliF of MG1655 (Figure 3A,B and Figure S6). Conservation of amino acid motifs predicts enzymatic activity for the proteins (Figure S6). In addition, truncated versions of the protein are found on several plasmids (Figure S6). A second GGDEF protein encoded by plasmids is with 83, 35 and 35% identity over the entire length of the protein homologous to the Globin-CBS-GGDEF protein DosC, also one of the *E. coli* MG1655 core genome GGDEF proteins.

**Figure 4:**
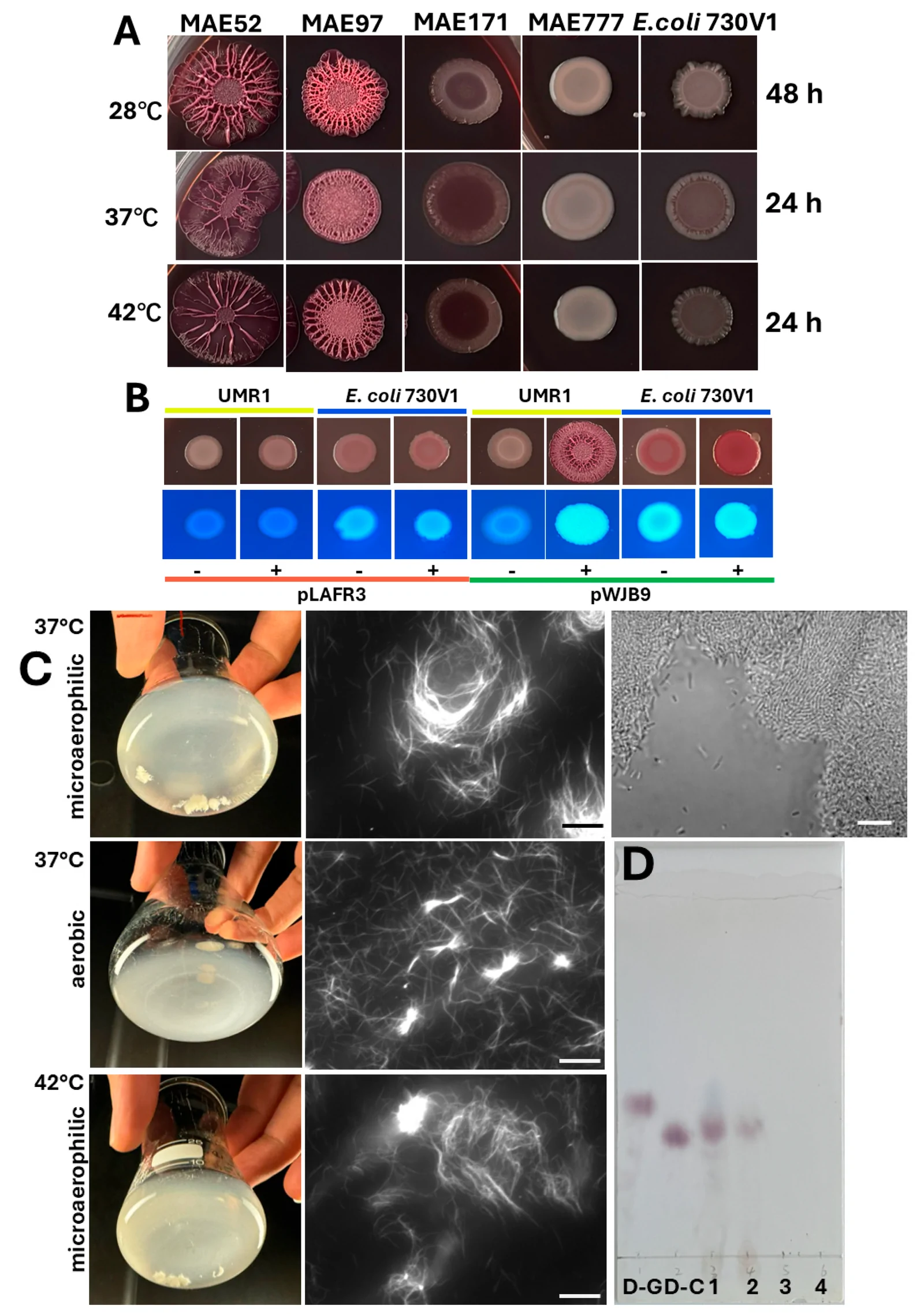
Biofilm formation and analysis of cellulose production in *E. coli* 730V1 encoding a *bcs* EcType 4 gene cluster on plasmid pU1. (**A**) Colony morphology of *E. coli* 730V1 grown on Congo red agar plates at three relevant temperatures representing environmental, human body and cattle gut temperature. *S. typhimurium* MAE52, an ATCC 14028 derivative (Nal^r^, P*csgD1*), and its curli (MAE97), cellulose (MAE171), and cellulose/curli-negative (MAE777) mutants served as references for expression of extracellular matrix components. Side length of Congo red plate: 2.3 cm. (**B**) Colony morphology of *E. coli* 730V1 Nal^r^ expressing the diguanylate cyclase AdrA from the pLAFR3 plasmid under the control of the p*BAD* promoter (pWJB9) induced by L-arabinose [20]. *S. typhimurium* UMR1 containing pLAFR3 and pWJB9 served as reference. Note that the rdar morphotype of *E. coli* 730V1 Nal^r^ is more pronounced than wild-type *E. coli* 730V1. Cells were grown on a Congo red agar plate at 28 °C for 48 h. Side length of Congo red plate: 2.3 cm. Side length of Calcofluor white plate: 1.9 cm. (**C**) Cell aggregation and Calcofluor white binding of *E. coli* 730V1 grown in M9 minimal medium with 0.4% glucose as carbon source at indicated oxygen pressure and temperature for 24 h. (**D**) Product analysis after cellulase digestion of KOH extracted exopolysaccharide from *E. coli* 730V1 grown in M9 minimal medium with 0.4% glucose as carbon source. Standard, D-G, D-glucose; D-C, cellobiose; 1, microcrystalline cellulose (Sigma-Aldrich) digested with *Trichoderma viride* cellulase; 2, KOH-extracted exopolysaccharide from *E. coli* 730V1 digested with *T. viride* cellulase; 3, *T. viride* cellulase; 4, undigested KOH-extracted exopolysaccharide from *E. coli* 730V1.

Among the EAL domain proteins, the SP (signal peptide)-CHASE9-xCache-TM-HAMP-xGGDEF-EAL phosphodiesterase YliE is most frequently found on *bcs* EcType 2-, EcType 3- and EcType 4-bearing plasmids and on the chromosome in several variants (Figure 4C,D and Figure S6). The three major YliE clades of full-length proteins are 35, 37 and 28% identical to YliE of *E. coli* MG1655 and were previously identified as plasmid-located [65]. As on the *E. coli* MG1655 chromosome, *yliE* is located adjacent to *yliF* at some of the *bcs* gene clusters. Besides YliE, variants of the EAL protein PdeW previously identified on plasmids are plasmid-located. In addition, variants of UrfM (QGGX6) originally identified on transposon Tn21 and a novel GGDEF protein with an unidentified N-terminal signaling domain are found on *bcsA*-bearing plasmids.

### 3.7. Other Bacterial Species Also Harbor Cellulose Biosynthesis Gene Clusters on Plasmids

When assessing the occurrence of the BcsA cellulose synthase on plasmids, we noticed that plasmids contained by species isolates other than *E. coli* also encode cellulose synthases with proteins closely or distantly related to the *E. coli* core genome cellulose synthase BcsA1 (Figure S1, Table S1). Thus, cellulose biosynthesis is subject to horizontal gene transfer in a larger context. Most of the BcsA-bearing plasmids were harbored by *Rhizobium* spp. and *Sino-*(*Para-* and *Neo-*)*rhizobium* spp. isolates, alpha-proteobacterial plant symbionts known to exploit cellulose production for bacterial host interactions [9,66]. *Ensifier* spp. *Azospirillium* spp, *Caballeronia* spp. and *Klebsiella* spp. isolates are also frequently found to harbor BcsA-bearing plasmids.

### 3.8. Identification of Bacterial Species That Harbor More than One Cellulose Biosynthesis Operon

*Komagataeibacter* spp., *Proteus* spp. and *Klebsiella* spp. are well known to harbor more than one cellulose biosynthesis gene cluster [5,14,25]. In fact *Komagataeibacter* spp. can harbor up to four distinct *bcs* gene clusters [67]. Some of these cellulose synthases can be highly similar, indicating gene duplication rather than horizontal gene transfer. As BcsA cellulose synthases are often found on plasmids, we were wondering whether those genes can become manifested on chromosomes as second cellulose biosynthesis entities. Using the Uniprot protein database, we investigated which species (or distinct isolates of a species) harbor more than one cellulose biosynthesis. In particular, *Klebsiella* spp. and *Pseudomonas* spp. isolates can harbor more than one cellulose synthase often with low similarity indicating horizontal transfer from a phylogenetically unrelated species (Figure S7A). Surprisingly though, the second cellulose biosynthesis gene cluster is inserted immediately adjacent to the first cellulose biosynthesis gene cluster in *K. pneumoniae* isolates and can also be found as a third cellulose biosynthesis gene cluster on a plasmid in *K. pneumoniae* WS29-1 (Figure S7B).

### 3.9. Rdar Biofilm Formation and Cellulose Biosynthesis of E. coli 730V1 Harboring a bcs4 Cellulose Biosynthesis Operon

Remarkably, a number of *E. coli* strains bear a second or an alternative *bcs* gene cluster. We identified the *Bos taurus* derived the temperature-tolerant isolate *E. coli* 730V1 [26] as a strain bearing the EcType 4 cellulose biosynthesis gene cluster *bcsOQABCDZ bcsEFG* on the pU1 plasmid with its chromosomal *bcs* EcType 1 operon deleted (Figure 2B). The deletion of almost the entire chromosomal *bcs* EcType 1 gene cluster was confirmed by PCR (Table 1 and Table S3, Figure 2B). In *E. coli* and *S. typhimurium*, the exopolysaccharide cellulose is conventionally produced as an extracellular matrix component of environmental and host-associated biofilms [68]. For example, concomitantly with amyloid fimbriae, cellulose is one of the two major extracellular matrix components of the agar-grown rdar (red, dry and rough) colony biofilm which impacts host and plant associations [69]. Rdar biofilm formation is highly regulated in most *E. coli* and *S. typhimurium* strains being expressed at ambient temperature below 30 °C, but semi-constitutive biofilm formation at human body temperature can occur, in particular, in *E. coli* [27,50,70]. *Escherichia coli* 730V1 is derived from processed meat with a history of heat intervention for decontamination and is highly thermotolerant due to the presence of the transmissible locus of stress tolerance tLST. Consequently, we assessed rdar biofilm formation and cellulose production of *E. coli* 730V1 not only at 28 °C and 37 °C but also at 42 °C and 45 °C on Congo red and Calcofluor white (Fluorescence Brightener 28) agar plates. In this context, we compared *E. coli* 730V1 with the model *S. typhimurium* MAE52 (an ATCC 14028 derivative) and its derivatives [27].

We found *E. coli* 730V1 to express a weak rdar morphotype at 37 °C and 42 °C but not 28 °C on Congo red agar plates under atmospheric oxygen (Figure 4A,B). In contrast, the plate-grown strains substantially bound to Calcofluor white (Figure 4B). This dye-binding pattern is not entirely consistent with conventional cellulose expression, as the fluorochrome Calcofluor white is also known to bind to 1,3 and 1,4 β-glucans other than cellulose. Furthermore, the strain showed strong cell aggregation in M9 minimal medium at 37 °C and 42 °C when grown under microaerophilic conditions, while Calcofluor white binding was also observed under aerobic conditions without strong aggregation (Figure 4C). No substantial cell aggregation was observed in LB-NS medium at the two distinct oxygen pressures.

As the rdar morphotype and Calcofluor white binding are not absolutely informative for cellulose expression, we expressed the diguanylate cyclase AdrA in *E. coli* 730V1 (Figure 4B). As a control, AdrA was expressed in *S. typhimurium* UMR1. In order to enable the conjugation of the *adrA* gene, we selected a nalidixic acid-resistant variant of *E. coli* 730V1. Of note, *E. coli* 730V1 Nal^r^ showed a slightly upregulated rdar morphotype (Figure S8A). Upon acquisition of the *adrA*-bearing plasmid pLAFR3, we observed elevated Congo red binding indicative for cellulose expression in *E. coli* 730V1 as for the *S. typhimurium* UMR1 control strain. Further, we isolated crystalline cellulose with KOH (obtaining crystalline and amorphous cellulose) and the Updegraff reagent (obtaining crystalline cellulose) from *E. coli* 730V1 grown under microaerophilic conditions in M9 minimal medium (Figure S8B) and subjected it to cellulase digestion. Digestion of KOH digested cellulose isolated from *E. coli* 730V1 and microcrystalline cellulose with cellulase obtained products that run at the height of cellobiose (Figure 4D). We conclude that at least a fraction of the extracellular matrix produced in M9 minimal medium is cellulose.

**Figure 5:**
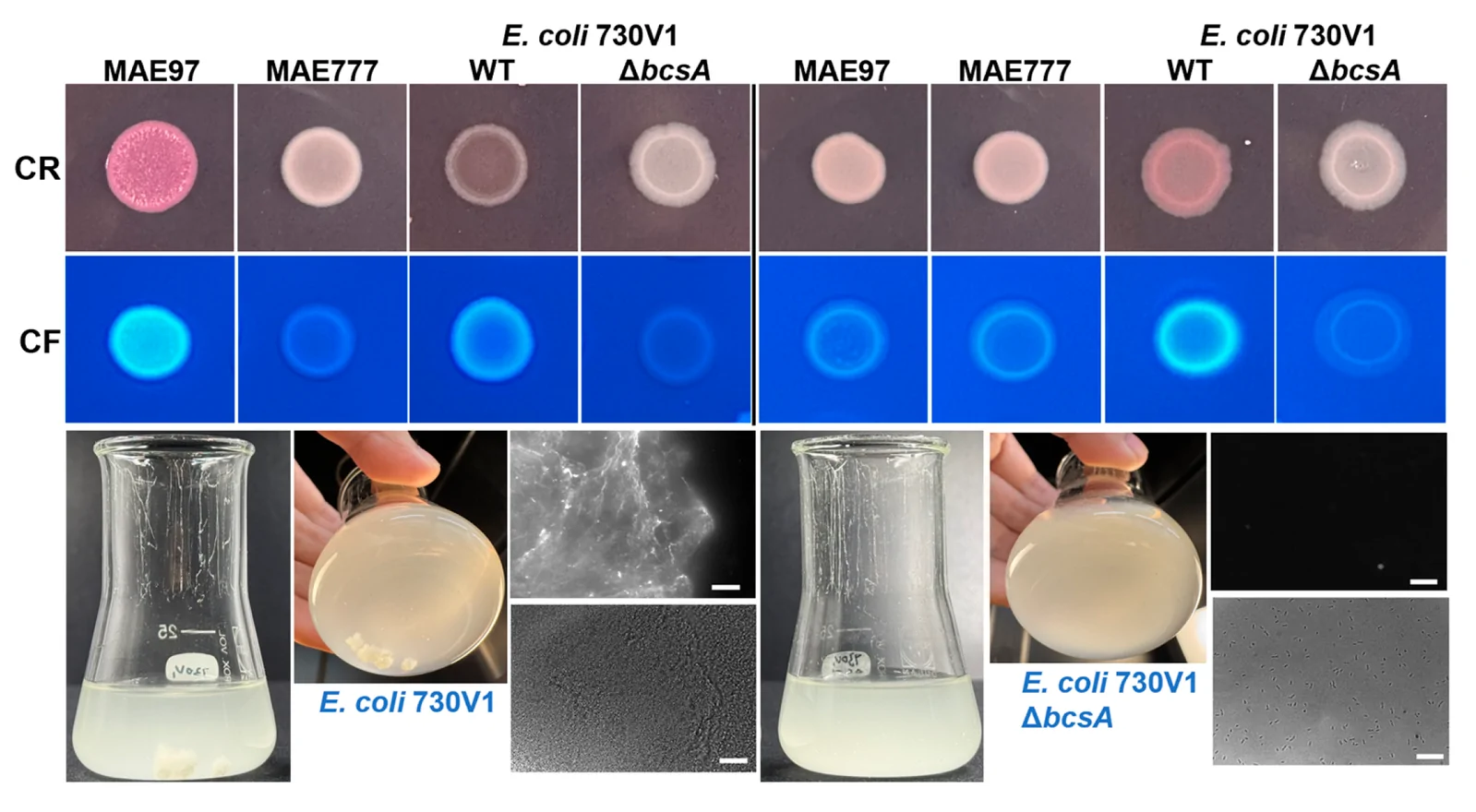
Phenotypes of the Δ*bcsA4* mutant of *E. coli* 730V1 on Congo red agar plates and in M9 minimal medium + 0.4% glucose. Upper row: colony morphotypes on CR Congo red agar plates at 42 (**left**) and 45 °C (**right**). Second row: Calcofluor white (CF) binding. Positive control: MAE97 and negative control MAE777. Note that MAE97 produced cellulose at 42 but not at 45 °C. Last row: cell aggregation in M9 minimal medium + 0.4% glucose grown under microaerophilic conditions at 37 °C. Size bar: 10 µm.

In order to unambiguously demonstrate that the multicellular behavior of *E. coli* 730V1, in particular the cell aggregation in M9 minimal medium, is due to the biosynthesis of cellulose, we replaced the *bcsA4* cellulose synthase gene by a kanamycin and chloramphenicol resistance cassette. Assessment of the phenotype of the *bcsA4* mutant on Congo red agar plates and in M9 minimal medium showed that dye-binding properties and cell aggregation have been completely abolished (Figure 5). Microscopic observation demonstrated that the wild-type produced Calcofluor white binding aggregates were not present in the Δ*bcsA4* mutant (Figure 4C and Figure 5). Consequently, the dye-binding and aggregation behavior of strain *E. coli* 730V1 is due to cellulose biosynthesis.

### 3.10. The Unconventional Cellulose Biosynthesis Gene Cluster Is Restricted to E. coli 730V1 in Meat-Derived Isolates

Concomitantly with *E. coli* 730V1, another genetically distinct *E. coli* isolate, 730-2, was derived from the same meat sample [26] and tested for the presence of the *bcs* EcType 4 gene cluster. The PCR assay was positive for the core genome *bcsA1* and *bcsB1* genes, but not for the *bcsA4* gene (Table S3). The biofilm phenotype as assessed on Congo red agar plates was also distinct (Figure S9A). While strain *E. coli* 730V1 displayed enhanced colony biofilm (pdar morphotype indicative for cellulose expression) at 42 °C after plate incubation in a microaerophilic atmosphere with 10% CO_2_ as compared to aerobic incubation and microaerophilic incubation at 37 °C, *E. coli* 730-2 displayed a bdar colony morphotype only after plate incubation under aerobic conditions at 37 °C. During incubation in liquid medium, *E. coli* 730V1 showed substantial cell aggregation under microaerophilic conditions created by two different means (gas pack creating a 5% O_2_/10% CO_2_ atmosphere or microaerophilic growth achieved by level of flask filling combined with low shaking speed). Different types of aggregates were observed, visible and uniform larger granules under 5% O_2_/10% CO_2_ atmospheric growth versus more pronounced irregular, larger aggregates under microaerophilic growth (Figure S9B,C). No aggregates were observed under aerobic conditions and in rich (LB-NS) medium under aerobic and microaerophilic conditions (Figure S9B–D). *E. coli* 730-2 did not aggregate under all tested conditions. Table S4 summarizes biofilm formation under the various growth conditions.

Furthermore, the screening of nine additional selected meat-derived *E. coli* isolates [26] did not indicate the presence of the *bcsA4B4* cellulose synthase genes. Of note, three of the strain did not test positive for *bcsA1* suggesting that loss of the core genome cellulose biosynthesis gene cluster might occur more often in isolates from meat-derived samples (Table S3).

## 4. Discussion

The exopolysaccharide cellulose and structurally similar poly-glucans are widespread extracellular matrix components of microbial biofilms [15,17,71]. The 1,4-β-D-glucan-based cellulose and its covalently modified derivatives are produced throughout the bacterial phylogenetic tree. Cellulose producers include environmental bacteria, such as thermophilic bacteria and cyanobacteria in microbial mats, and pathogens such as *S. typhimurium*. In today’s human pathogens, cellulose production has been intriguingly re-functionalized to trade acute virulence and cytotoxicity (also within immune cells) versus environmental survival and persistence in the host [5,7,19,20,51]. Cellulose and derivatives are thus multipurpose macromolecules that can protect against environmental stresses such as UV radiation and chlorine exposure [54,72]. As an anti-virulence factor, cellulose biosynthesis prevents adhesion and invasion of bacteria into epithelial cell lines and restricts the proliferation of *Salmonella* in professional macrophages [19,68]. Consequently, enhanced invasiveness and acute virulence of pathogens are associated with diminished or abolished cellulose production [21,22]. However, in which ecological context a second/alternative cellulose biosynthesis operon/gene cluster can provide an advantage for *E. coli* remains to be investigated. The differential expression of the cellulose synthase and cellulose biosynthesis and altered physicochemical properties of the synthesized cellulose macromolecule might play a role. Indeed, under the investigated experimental conditions, cellulose is predominantly produced at elevated temperature and displays as amorphous upon Calcofluor white staining (Figure 4).

In bacteria, cellulose exists not only as crystalline macrofibrills but as bundles of amorphous 1,4-β-D-glucan chains. In several instances, the glucose subunits have been found to be covalently modified such as in the case of type II cellulose biosynthesis operons, where glucose upon incorporation into the nascent glucan chain is covalently modified by the addition of phosphoethanolamine to the C6 carbon [11,13]. The multifunctional alkaline phosphatase superfamily member BcsG not only catalyzes this transfer reaction from the most abundant phospholipid phosphoethanoleamine to glucose by its periplasmic catalytic domain, but also stabilizes the BcsA cellulose synthase via its N-terminal transmembrane helices [11,13]. The presence of solely the *bcsABC* gene cluster as a second copy suggests that those *E. coli* strains can produce unmodified crystalline cellulose and that the cellulose synthase BcsA does not require stabilization by BcsG. In support for this hypothesis, BcsA2 and BcsA3 lack the first N-terminal approx. 150 amino acids and the extended C-terminus (Figure S1) that have been shown to interact with trimeric BcsG [8,73]. However, even type Ib BcsA4, with which BcsG variants of type IIa are co-localized (Figure 1 and Figure S4), lack the N- and C-terminal BcsG interacting sequences, suggesting that BcsG interacts with BcsA4 cellulose synthases in an alternative way.

Despite the obvious impact of cellulose biosynthesis in the protection against environmental stresses, there is an open question as to why bacterial species possess more than one cellulose biosynthesis gene cluster, as achieving a higher cellulose production might be limited by physiological and metabolic constraints. Another open question is to which extent and, if so, how these clusters are horizontally transferred and duplicated. The fruit-degrading genus *Komagataeibacter* spp., including *K. xylinus*, are model organisms for cellulose biosynthesis and are the only bacterial genus that produces economically relevant amounts of cellulose; up to four cellulose biosynthesis operons have been identified per genome [67]. Those cellulose biosynthesis operons are usually all of type I but associated with different accessory gene products. However, one cellulose biosynthesis operon seems to be dominant in the production of cellulose [25]. Our data suggest that type I cellulose biosynthesis gene clusters are encoded by *Klebsiella* spp. together with type II gene clusters. Perhaps the occurrence of the two different types of cellulose biosynthesis gene clusters encoded by one genome led to the acquisition of type II genes by type I gene clusters.

The two homologous cellulose biosynthesis gene clusters in *Klebsiella* and *Proteus* spp. show only low protein similarity and even belong to different types (Figure S7; [14]). One can hypothesize that one of these cellulose biosynthesis clusters has been horizontally transferred and subsequently integrated into the chromosome. The impact of multiple cellulose biosynthesis operons in these bacterial species is, however, not that obvious. For example, although *K. pneumoniae* possesses two co-localized cellulose biosynthesis gene clusters, *K. pneumoniae* conventionally displays a mucoid colony type under laboratory conditions. Only rarely has cellulose expressing pdar colony morphotype biofilms been observed in *Klebsiella* [17]. In particular, recently, cellulose producers have caused outbreaks in hospitals in China due to their high biofilm formation capacity [74]. Of note, although possessing only one GGDEF diguanylate cyclase with cyclic di-GMP required for post-translational activation of cellulose biosynthesis, *Proteus* spp. possess two cellulose biosynthesis gene clusters. However, cellulose biosynthesis in *Proteus* spp. has not been observed, even upon the overexpression of the co-localized functional diguanylate cyclase [14].

The situation seems to be somewhat different in the species *E. coli*. Already present in the common ancestor of *Salmonella* and *E. coli*, the chromosomal location of the cellulose biosynthesis operon is highly conserved. But although diverse second cellulose biosynthesis gene clusters have been observed on plasmids in this work, a second cellulose biosynthesis gene cluster has not been widely established on the chromosome or on plasmids in this species. Thus, although potentially providing a temporal and spatial niche advantage, the acquisition of a second operon does not seem to be evolutionary favorable in *E. coli* on the population-wide level. In this context, it is worth mentioning that mobile group II intron retroelements [75] often colocalize with horizontally transferred cellulose biosynthesis operons.

In contrast, the poly-N-acetyglucoseamine (PNAG) biosynthesis operon, synthesizing a second major exopolysaccharide of *E. coli* as an extracellular matrix component of biofilms and originally characterized in *E. coli* K-12 [76], is not present in all *E. coli* strains. Furthermore the operon has a variable chromosomal position in the different strains [50]. Thus, we hypothesized that the PNAG biosynthesis operon can be equally subject to horizontal gene transfer. Indeed, BLAST search using the PNAG synthase PgaC showed that the *pgaABCD* operon can be found on *E. coli* mobile elements such as plasmids (Figure S10; Table S5). In addition, *pgaABCD* operons that are highly similar to the chromosomally encoded PNAG operon are found on phage-like mobile elements suggesting their recent mobilization from the chromosome in different *E. coli* strains. In conclusion, even seemingly conserved biofilm components are more mobile than previously thought, but they are not necessarily established population-wide in the chromosomal context.

The major regulatory mechanism to promote enhanced cellulose biosynthesis in *E. coli* is the post-translational relief of inhibition of catalytic activity by the binding of the second messenger cyclic di-GMP to the C-terminal PilZ domain of bacterial cellulose synthases. Thereby, cellulose biosynthesis can be activated by cyclic di-GMP produced by the diguanylate cyclase AdrA via a regulatory pathway induced by the transcriptional regulator CsgD and independently of CsgD by the diguanylate cyclase DgcQ and unknown diguanylate cyclases, in different *E. coli* isolates [20,77,78]. Of note, we found that cyclic di-GMP turnover proteins, GGDEF diguanylate cyclases or phosphodiesterases are frequently co-encoding with the alternative cellulose biosynthesis gene cluster in the chromosome and in plasmids (Figure 1B, Figure 2B and Figure S1). Thereby, the homologs of core genome-encoded diguanylate cyclases YliF and DosC are most frequently associated with alternative *bcs* gene clusters on plasmids and chromosomes. Core genome DosC forms a complex with the phosphodiesterase DosP and the cyclic di-GMP receptor polynucleotide phosphorylase (PNPase) for oxygen dependent cyclic di-GMP control [79,80]. While DosC (alternative name YddV) activates poly-N-acetyl-glucoseamine biosynthesis and curli production on the transcriptional level [81,82], neither YliF nor DosC have been previously reported to stimulate cellulose biosynthesis in the native chromosomal context. Those cyclic di-GMP synthesizing proteins might be responsible for cellulose biosynthesis, including cell aggregation under specific environmental conditions (Figure 5; Table S4). Of note, those diguanylate cyclases have a low similarity over the entire length of the protein compared to their core genome counterparts and are encoded in a different genomic context suggesting distinct regulatory and catalytic features. A BLAST search indicated these homologous proteins, equally as the BcsA2 and BcsA3 cellulose synthases, to be restricted to the *Escherichia* genus. One hypothesis is that mobilization on plasmids and horizontal transfer promotes sequence diversification within the genus. Alternatively, conservation of domain structure associated with diversification has occurred in distantly related (unidentified) species and genes were subsequently mobilized.

## 5. Conclusions

Besides the loss of cellulose biosynthesis associated with enhanced invasiveness and virulence of *E. coli* isolates, a second cellulose biosynthesis gene cluster can also be acquired with occasional loss of the core genome-encoded cellulose biosynthesis gene cluster. While such a cellulose biosynthesis cluster can be functional, as shown upon post-translational activation by cyclic di-GMP and *bcsA4* mutant construction in the meat-derived strain *E. coli* 730V1, the evolutionary drivers of acquisition and, potentially, maintenance of a second (and/or alternative) cellulose biosynthesis gene cluster have not yet been unraveled. Their infrequent occurrence points to the fact that they provide advantages in specific genetic backgrounds and/or in specific ecological niches. As such, the factors that efficiently promote horizontal gene and plasmid transfer will provide clues for their mode of mobilization and dissemination. Assessment of the role(s) of an alternative or a second cellulose biosynthesis gene cluster in protection against environmental stresses, as anti-virulence factors and possessing an alternative expression profile, will provide clues about additive or complementary functionality. Investigation into whether BcsA4 interacts with the co-localized BcsG4 will provide alternative protein–protein interaction modes that eventually lead to phosphoethanolamine-modified cellulose. Equally, can the loss of the *E. coli* 730V1 pU1 plasmid encoding the *bcs* EcType 4 cellulose biosynthesis gene cluster indicate the detrimental environmental conditions that counteract cellulose biosynthesis.

## Figures and Tables

**Table 1:** Primers and plasmids used for gene deletion and strain control in this study.

Primer Name	Sequence 5′-3′ *	Reference
F-DEL-BcsA-730v1	atgaaaaagtctcttttttggttgctggcgctggtgctgtTTGAGCGATTGTGTAGGCTG	This study
R-DEL-BcsA-730v1	tcataacgtcccatcctcctgcgccgttttcgccactgttGGGAATTAGCCATGGTCCAT	This study
BcsA-control-F	GTATTCATATTGGCGATGGCA	This study
BcsA-control-R	GCCCGGTTCTTCACTGTGTA	This study
730V1_bcsG_f	AAATCTTGCCATCAAGAACGCGAAC	This study
730V1_bcsC_out_r	AAATTGTCTCCCCCCGGAAATC	This study
**Plasmid**	**Description**	**Reference**
pKD4	Template plasmid for λ Red mediated recombination, Km^r^, Amp^r^	
pKD88	pKD46 derivative, Cm^r^	This study
pBAD28	Template plasmid for amplification of the chloramphenicol resistance cassette for cloning into pKD46	This study

* Lowercase letter, nucleotide sequence homologous to the 5′ and 3′ ends of the *bcsA* gene.

## Data Availability

The original contributions presented in this study are included in the article/Supplementary Material. Further inquiries can be directed to the corresponding author.

## Supplementary Materials

The following supporting information can be downloaded at https://www.mdpi.com/article/10.3390/microorganisms14091932/s1: Figure S1: *E. coli* encoded cellulose synthases in the phylogenetic context. Figure S2: Representative plasmids harboring the different EcTypes *bcsA2-4* cellulose biosynthesis gene clusters and cyclic di-GMP turnover protein genes. Figure S3: Size distribution of BcsA-bearing plasmids versus all plasmids of *E. coli*, *Shigella* and *Escherichia* spp. Figure S4: Phylogenetic context of cellulose biosynthesis proteins from *E. coli*. Figure S5: Domain structure and length of GGDEF and EAL domain proteins encoded by BcsA encoding *E. coli* plasmids. Figure S6: Alignment of cyclic di-GMP turnover proteins associated with novel cellulose biosynthesis gene clusters. Figure S7: Phylogenetic relationship and arrangement of paralogous and xenologous BcsA cellulose synthases compared to core genome cellulose synthases encoded by individual isolates of a species. Figure S8: Characterization of cellulose biosynthesis in *E. coli* 730V1 Nal^r^. Figure S9: Comparison of cellulose production and biofilm phenotypes between *E. coli* 730V1 and 730-2 isolated from the same meat sample. Figure S10: Genomic context of mobile element encoded poly-N-acetyl-glucosamine *pgaABCD* biosynthesis gene clusters [39,83,84,85,86]. Table S1: List of reference plasmids bearing alternative BcsA cellulose synthases. Table S2: Cyclic di-GMP turnover proteins found on BcsA-bearing plasmids. Table S3: Presence of *bcsA1* and *bcsA4* cellulose synthase genes in selected meat-derived *E. coli* strains. Table S4: Summary of biofilm phenotypes for *E. coli* 730V1. Table S5: List of *pgaABCD* encoding mobile plasmidic elements in *E. coli.*

## Abbreviations

The following abbreviations are used in this manuscript:

Bcs	Bacterial cellulose biosynthesis/bacterial cellulose synthase
bdar	Brown, dry and rough
bas	Brown and smooth
pdar	Pink, dry and rough
rdar	Red, dry and rough
ras	Red and smooth
saw	Smooth and white
ST	Sequence type

## References

[1] Klemm D., Heublein B., Fink H.P., Bohn A. (2005). Cellulose: Fascinating biopolymer and sustainable raw material. Angew. Chem. Int. Ed. Engl..

[2] Fugelstad J., Bouzenzana J., Djerbi S., Guerriero G., Ezcurra I., Teeri T.T., Arvestad L., Bulone V. (2009). Identification of the cellulose synthase genes from the Oomycete *Saprolegnia monoica* and effect of cellulose synthesis inhibitors on gene expression and enzyme activity. Fungal Genet. Biol..

[3] Nakashima K., Yamada L., Satou Y., Azuma J., Satoh N. (2004). The evolutionary origin of animal cellulose synthase. Dev. Genes. Evol..

[4] Pedersen G.B., Blaschek L., Frandsen K.E.H., Noack L.C., Persson S. (2023). Cellulose synthesis in land plants. Mol. Plant.

[5] Römling U., Galperin M.Y. (2015). Bacterial cellulose biosynthesis: Diversity of operons, subunits, products, and functions. Trends Microbiol..

[6] Scott W., Lowrance B., Anderson A.C., Weadge J.T. (2020). Identification of the *Clostridial* cellulose synthase and characterization of the cognate glycosyl hydrolase, CcsZ. PLoS ONE.

[7] Ahmad I., Rouf S.F., Sun L., Cimdins A., Shafeeq S., Le Guyon S., Schottkowski M., Rhen M., Römling U. (2016). BcsZ inhibits biofilm phenotypes and promotes virulence by blocking cellulose production in *Salmonella enterica* serovar Typhimurium. Microb. Cell Fact..

[8] Verma P., Ho R., Chambers S.A., Cegelski L., Zimmer J. (2024). Insights into phosphoethanolamine cellulose synthesis and secretion across the Gram-negative cell envelope. Nat. Commun..

[9] Robledo M., Rivera L., Jimenez-Zurdo J.I., Rivas R., Dazzo F., Velazquez E., Martinez-Molina E., Hirsch A.M., Mateos P.F. (2012). Role of *Rhizobium* endoglucanase CelC2 in cellulose biosynthesis and biofilm formation on plant roots and abiotic surfaces. Microb. Cell Fact..

[10] Menendez E., Robledo M., Jimenez-Zurdo J.I., Velazquez E., Rivas R., Murray J.D., Mateos P.F. (2019). Legumes display common and host-specific responses to the rhizobial cellulase CelC2 during primary symbiotic infection. Sci. Rep..

[11] Thongsomboon W., Serra D.O., Possling A., Hadjineophytou C., Hengge R., Cegelski L. (2018). Phosphoethanolamine cellulose: A naturally produced chemically modified cellulose. Science.

[12] Spiers A.J., Bohannon J., Gehrig S.M., Rainey P.B. (2003). Biofilm formation at the air-liquid interface by the *Pseudomonas fluorescens* SBW25 wrinkly spreader requires an acetylated form of cellulose. Mol. Microbiol..

[13] Sun L., Vella P., Schnell R., Polyakova A., Bourenkov G., Li F., Cimdins A., Schneider T.R., Lindqvist Y., Galperin M.Y. (2018). Structural and Functional Characterization of the BcsG Subunit of the Cellulose Synthase in *Salmonella typhimurium*. J. Mol. Biol..

[14] Liu Y., Lee C., Li F., Trcek J., Bähre H., Guo R.T., Chen C.C., Chernobrovkin A., Zubarev R., Römling U. (2020). A Cyclic di-GMP Network Is Present in Gram-Positive *Streptococcus* and Gram-Negative *Proteus* Species. ACS Infect. Dis..

[15] Perez-Mendoza D., Rodriguez-Carvajal M.A., Romero-Jimenez L., Farias Gde A., Lloret J., Gallegos M.T., Sanjuan J. (2015). Novel mixed-linkage beta-glucan activated by c-di-GMP in *Sinorhizobium meliloti*. Proc. Natl. Acad. Sci. USA.

[16] Chang S.C., Kao M.R., Saldivar R.K., Diaz-Moreno S.M., Xing X., Furlanetto V., Yayo J., Divne C., Vilaplana F., Abbott D.W. (2023). The Gram-positive bacterium *Romboutsia ilealis* harbors a polysaccharide synthase that can produce (1,3;1,4)-beta-D-glucans. Nat. Commun..

[17] Zogaj X., Nimtz M., Rohde M., Bokranz W., Römling U. (2001). The multicellular morphotypes of *Salmonella typhimurium* and *Escherichia coli* produce cellulose as the second component of the extracellular matrix. Mol. Microbiol..

[18] Bokranz W., Wang X., Tschape H., Römling U. (2005). Expression of cellulose and curli fimbriae by *Escherichia coli* isolated from the gastrointestinal tract. J. Med. Microbiol..

[19] Pontes M.H., Lee E.J., Choi J., Groisman E.A. (2015). *Salmonella* promotes virulence by repressing cellulose production. Proc. Natl. Acad. Sci. USA.

[20] Römling U., Rohde M., Olsen A., Normark S., Reinköster J. (2000). AgfD, the checkpoint of multicellular and aggregative behaviour in *Salmonella typhimurium* regulates at least two independent pathways. Mol. Microbiol..

[21] Nhu N.T.K., Rahman M.A., Goh K.G.K., Kim S.J., Phan M.D., Peters K.M., Alvarez-Fraga L., Hancock S.J., Ravi C., Kidd T.J. (2024). A convergent evolutionary pathway attenuating cellulose production drives enhanced virulence of some bacteria. Nat. Commun..

[22] Singletary L.A., Karlinsey J.E., Libby S.J., Mooney J.P., Lokken K.L., Tsolis R.M., Byndloss M.X., Hirao L.A., Gaulke C.A., Crawford R.W. (2016). Loss of Multicellular Behavior in Epidemic African Nontyphoidal *Salmonella enterica* Serovar Typhimurium ST313 Strain D23580. mBio.

[23] Sakellaris H., Hannink N.K., Rajakumar K., Bulach D., Hunt M., Sasakawa C., Adler B. (2000). Curli loci of *Shigella* spp. Infect. Immun..

[24] Chanin R.B., Nickerson K.P., Llanos-Chea A., Sistrunk J.R., Rasko D.A., Kumar D.K.V., de la Parra J., Auclair J.R., Ding J., Li K. (2019). *Shigella flexneri* Adherence Factor Expression in In Vivo-Like Conditions. mSphere.

[25] Saxena I.M., Brown R.M. (1995). Identification of a second cellulose synthase gene (*acsAII*) in *Acetobacter xylinum*. J. Bacteriol..

[26] Guragain M., Brichta-Harhay D.M., Bono J.L., Bosilevac J.M. (2021). Locus of Heat Resistance (LHR) in Meat-Borne *Escherichia coli*: Screening and Genetic Characterization. Appl. Environ. Microbiol..

[27] Römling U., Sierralta W.D., Eriksson K., Normark S. (1998). Multicellular and aggregative behaviour of *Salmonella typhimurium* strains is controlled by mutations in the *agfD* promoter. Mol. Microbiol..

[28] Simm R., Morr M., Kader A., Nimtz M., Römling U. (2004). GGDEF and EAL domains inversely regulate cyclic di-GMP levels and transition from sessility to motility. Mol. Microbiol..

[29] Datsenko K.A., Wanner B.L. (2000). One-step inactivation of chromosomal genes in *Escherichia coli* K-12 using PCR products. Proc. Natl. Acad. Sci. USA.

[30] Altschul S.F., Gish W., Miller W., Myers E.W., Lipman D.J. (1990). Basic local alignment search tool. J. Mol. Biol..

[31] Higgins D.G., Sharp P.M. (1988). CLUSTAL: A package for performing multiple sequence alignment on a microcomputer. Gene.

[32] Sievers F., Higgins D.G. (2021). The Clustal Omega Multiple Alignment Package. Methods Mol. Biol..

[33] Nicholas K.B., Nicholas H.B., Deerfield D.W. (1997). GeneDoc: Analysis and Visualization of Genetic Variation. EMBnet.news.

[34] Stothard P. (2000). The sequence manipulation suite: JavaScript programs for analyzing and formatting protein and DNA sequences. Biotechniques.

[35] Robert X., Gouet P. (2014). Deciphering key features in protein structures with the new ENDscript server. Nucleic Acids Res..

[36] Tamura K., Stecher G., Kumar S. (2021). MEGA11: Molecular Evolutionary Genetics Analysis Version 11. Mol. Biol. Evol..

[37] Minh B.Q., Schmidt H.A., Chernomor O., Schrempf D., Woodhams M.D., von Haeseler A., Lanfear R. (2020). IQ-TREE 2: New Models and Efficient Methods for Phylogenetic Inference in the Genomic Era. Mol. Biol. Evol..

[38] Cao L.Y., Yang Y.F., Zhang X., Chen Y.H., Yao J.W., Wang X., Xia J., Liu C.G., Yang S.H., Römling U. (2022). Deciphering Molecular Mechanism Underlying Self-Flocculation of *Zymomonas mobilis* for Robust Production. Appl. Environ. Microbiol..

[39] Sullivan M.J., Petty N.K., Beatson S.A. (2011). Easyfig: A genome comparison visualizer. Bioinformatics.

[40] Moller S., Croning M.D., Apweiler R. (2001). Evaluation of methods for the prediction of membrane spanning regions. Bioinformatics.

[41] Tsirigos K.D., Peters C., Shu N., Kall L., Elofsson A. (2015). The TOPCONS web server for consensus prediction of membrane protein topology and signal peptides. Nucleic Acids Res..

[42] Römling U. (2002). Molecular biology of cellulose production in bacteria. Res. Microbiol..

[43] Oehme D.P., Shafee T., Downton M.T., Bacic A., Doblin M.S. (2019). Differences in protein structural regions that impact functional specificity in GT2 family beta-glucan synthases. PLoS ONE.

[44] Xie Y., Li H., Luo X., Li H., Gao Q., Zhang L., Teng Y., Zhao Q., Zuo Z., Ren J. (2022). IBS 2.0: An upgraded illustrator for the visualization of biological sequences. Nucleic Acids Res..

[45] Omasits U., Ahrens C.H., Müller S., Wollscheid B. (2014). Protter: Interactive protein feature visualization and integration with experimental proteomic data. Bioinformatics.

[46] Carattoli A., Hasman H. (2020). PlasmidFinder and In Silico pMLST: Identification and Typing of Plasmid Replicons in Whole-Genome Sequencing (WGS). Methods Mol. Biol..

[47] Galata V., Fehlmann T., Backes C., Keller A. (2019). PLSDB: A resource of complete bacterial plasmids. Nucleic Acids Res..

[48] Grant J.R., Enns E., Marinier E., Mandal A., Herman E.K., Chen C.Y., Graham M., Van Domselaar G., Stothard P. (2023). Proksee: In-depth characterization and visualization of bacterial genomes. Nucleic Acids Res..

[49] Seemann T. (2014). Prokka: Rapid prokaryotic genome annotation. Bioinformatics.

[50] Cimdins A., Simm R., Li F., Lüthje P., Thorell K., Sjöling A., Brauner A., Römling U. (2017). Alterations of c-di-GMP turnover proteins modulate semi-constitutive rdar biofilm formation in commensal and uropathogenic *Escherichia coli*. Microbiologyopen.

[51] Gerstel U., Römling U. (2001). Oxygen tension and nutrient starvation are major signals that regulate *agfD* promoter activity and expression of the multicellular morphotype in *Salmonella typhimurium*. Environ. Microbiol..

[52] Updegraff D.M. (1969). Semimicro determination of cellulose in biological materials. Anal. Biochem..

[53] Humbel R., Collart M. (1975). Oligosaccharides in urine of patients with glycoprotein storage diseases: I. Rapid detection by thin-layer chromatography. Clin. Chim. Acta.

[54] Solano C., Garcia B., Valle J., Berasain C., Ghigo J.M., Gamazo C., Lasa I. (2002). Genetic analysis of *Salmonella enteritidis* biofilm formation: Critical role of cellulose. Mol. Microbiol..

[55] Morgan J.L., McNamara J.T., Zimmer J. (2014). Mechanism of activation of bacterial cellulose synthase by cyclic di-GMP. Nat. Struct. Mol. Biol..

[56] Pfeifer E., Moura de Sousa J.A., Touchon M., Rocha E.P.C. (2021). Bacteria have numerous distinctive groups of phage-plasmids with conserved phage and variable plasmid gene repertoires. Nucleic Acids Res..

[57] Kamal S.M., Cimdins-Ahne A., Lee C., Li F., Martin-Rodriguez A.J., Seferbekova Z., Afasizhev R., Wami H.T., Katikaridis P., Meins L. (2021). A recently isolated human commensal *Escherichia coli* ST10 clone member mediates enhanced thermotolerance and tetrathionate respiration on a P1 phage-derived IncY plasmid. Mol. Microbiol..

[58] Fang X., Ahmad I., Blanka A., Schottkowski M., Cimdins A., Galperin M.Y., Römling U., Gomelsky M. (2014). GIL, a new c-di-GMP-binding protein domain involved in regulation of cellulose synthesis in enterobacteria. Mol. Microbiol..

[59] Wong H.C., Fear A.L., Calhoon R.D., Eichinger G.H., Mayer R., Amikam D., Benziman M., Gelfand D.H., Meade J.H., Emerick A.W. (1990). Genetic organization of the cellulose synthase operon in *Acetobacter xylinum*. Proc. Natl. Acad. Sci. USA.

[60] Sana T.G., Notopoulou A., Puygrenier L., Decossas M., Moreau S., Carlier A., Krasteva P.V. (2024). Structures and roles of BcsD and partner scaffold proteins in proteobacterial cellulose secretion. Curr. Biol..

[61] Jeon Y.J., Xun Z., Su P., Rogers P.L. (2012). Genome-wide transcriptomic analysis of a flocculent strain of *Zymomonas mobilis*. Appl. Microbiol. Biotechnol..

[62] Ross P., Weinhouse H., Aloni Y., Michaeli D., Weinberger-Ohana P., Mayer R., Braun S., de Vroom E., van der Marel G.A., van Boom J.H. (1987). Regulation of cellulose synthesis in *Acetobacter xylinum* by cyclic diguanylic acid. Nature.

[63] Schirmer T., Jenal U. (2009). Structural and mechanistic determinants of c-di-GMP signalling. Nat. Rev. Microbiol..

[64] Römling U., Liang Z.X., Dow J.M. (2017). Progress in Understanding the Molecular Basis Underlying Functional Diversification of Cyclic Dinucleotide Turnover Proteins. J. Bacteriol..

[65] Römling U., Cao L.Y., Bai F.W. (2023). Evolution of cyclic di-GMP signalling on a short and long term time scale. Microbiology.

[66] Napoli C., Dazzo F., Hubbell D. (1975). Production of cellulose microfibrils by *Rhizobium*. Appl. Microbiol..

[67] Ryngajllo M., Kubiak K., Jedrzejczak-Krzepkowska M., Jacek P., Bielecki S. (2019). Comparative genomics of the *Komagataeibacter* strains-Efficient bionanocellulose producers. Microbiologyopen.

[68] Lamprokostopoulou A., Römling U. (2022). Yin and Yang of Biofilm Formation and Cyclic di-GMP Signaling of the Gastrointestinal Pathogen *Salmonella enterica* Serovar Typhimurium. J. Innate Immun..

[69] Kai-Larsen Y., Lüthje P., Chromek M., Peters V., Wang X., Holm A., Kadas L., Hedlund K.O., Johansson J., Chapman M.R. (2010). Uropathogenic *Escherichia coli* modulates immune responses and its curli fimbriae interact with the antimicrobial peptide LL-37. PLoS Pathog..

[70] Cimdins-Ahne A., Naemi A.O., Li F., Simm R., Römling U. (2023). Characterisation of Variants of Cyclic di-GMP Turnover Proteins Associated with Semi-Constitutive rdar Morphotype Expression in Commensal and Uropathogenic *Escherichia coli* Strains. Microorganisms.

[71] Bundalovic-Torma C., Whitfield G.B., Marmont L.S., Howell P.L., Parkinson J. (2020). A systematic pipeline for classifying bacterial operons reveals the evolutionary landscape of biofilm machineries. PLoS Comput. Biol..

[72] Williams W.S., Cannon R.E. (1989). Alternative Environmental Roles for Cellulose Produced by *Acetobacter xylinum*. Appl. Environ. Microbiol..

[73] Anso I., Zouhir S., Sana T.G., Krasteva P.V. (2024). Structural basis for synthase activation and cellulose modification in the *E. coli* Type II Bcs secretion system. Nat. Commun..

[74] Xiang Y., Zhu K., Min K., Zhang Y., Liu J., Liu K., Han Y., Li X., Du X., Wang X. (2024). Characterization of a *Salmonella enterica* serovar Typhimurium lineage with rough colony morphology and multidrug resistance. Nat. Commun..

[75] Lambowitz A.M., Zimmerly S. (2004). Mobile group II introns. Annu. Rev. Genet..

[76] Itoh Y., Rice J.D., Goller C., Pannuri A., Taylor J., Meisner J., Beveridge T.J., Preston J.F., Romeo T. (2008). Roles of *pgaABCD* genes in synthesis, modification, and export of the *Escherichia coli* biofilm adhesin poly-beta-1,6-N-acetyl-D-glucosamine. J. Bacteriol..

[77] Da Re S., Ghigo J.M. (2006). A CsgD-independent pathway for cellulose production and biofilm formation in *Escherichia coli*. J. Bacteriol..

[78] Monteiro C., Saxena I., Wang X., Kader A., Bokranz W., Simm R., Nobles D., Chromek M., Brauner A., Brown R.M. (2009). Characterization of cellulose production in *Escherichia coli* Nissle 1917 and its biological consequences. Environ. Microbiol..

[79] Fekete F.J., Weinert E.E. (2026). Roles of biological heme-based sensors of O_2_ in controlling bacterial behavior. J. Inorg. Biochem..

[80] Tuckerman J.R., Gonzalez G., Gilles-Gonzalez M.A. (2011). Cyclic di-GMP activation of polynucleotide phosphorylase signal-dependent RNA processing. J. Mol. Biol..

[81] Tagliabue L., Antoniani D., Maciag A., Bocci P., Raffaelli N., Landini P. (2010). The diguanylate cyclase YddV controls production of the exopolysaccharide poly-N-acetylglucosamine (PNAG) through regulation of the PNAG biosynthetic *pgaABCD* operon. Microbiology.

[82] Tagliabue L., Maciag A., Antoniani D., Landini P. (2010). The *yddV-dos* operon controls biofilm formation through the regulation of genes encoding curli fibers’ subunits in aerobically growing *Escherichia coli*. FEMS Immunol. Med. Microbiol..

[83] Collinson S.K., Emödy L., Müller K.H., Trust T.J., Kay W.W. (1991). Purification and characterization of thin, aggregative fimbriae from. Salmonella Enteritidis J. Bacteriol..

[84] UniProt C. (2025). UniProt: The Universal Protein Knowledgebase in 2025. Nucleic Acids Res..

[85] Uhlich G.A., Keen J.E., Elder R.O. (2001). Mutations in the *csgD* promoter associated with variations in curli expression in certain strains of *Escherichia coli* O157:H7. Appl. Environ. Microbiol..

[86] Apweiler R., Bairoch A., Wu C.H., Barker W.C., Boeckmann B., Ferro S., Gasteiger E., Huang H., Lopez R., Magrane M. (2004). Uniprot: The universal protein knowledgebase. Nucleic Acids Res..

