# Supplementary material for: Diverse Horizontally Transferred Cellulose Biosynthesis Gene Clusters in Escherichia coli Strains
Source: Microorganisms. 2026 Sep 1;14(9):1932. doi: 10.3390/microorganisms14091932 (PMC13609730; doi:10.3390/microorganisms14091932)
Supplement: Supplementary file 1 [file microorganisms-14-01932-s001.zip › microorganisms-4402296_SupplementaryMaterials-R2a.pdf]

Supplementary materials

Diverse horizontally transferred cellulose biosynthesis gene clusters in *Escherichia coli* strains

Seyedmohammad Hosseinpourlamardi[1,%], Shaiqa Labiba[1,%], Li Li[1,%], Ali Dadvar[1,%], Manita Guragain[2], Ute Römling[1,*]

[1]Department of Microbiology, Tumor and Cell Biology, Biomedicum, Karolinska Institutet, Stockholm, Sweden
[2]Characterization and Interventions for Foodborne Pathogens, Agricultural Research Service, United States Department of Agriculture, Wyndmoor, PA 19038, USA

[%], equal contribution.

*Correspondence: ute.romling@ki.se; Tel.: 0046-8-52487319

Content **page**

Supplementary Materials and Methods 4

Figures

Figure S1A-D
E. coli encoded cellulose synthases in the phylogenetic context. 5

Figure S2
Representative plasmids harboring the different EcTypes *bcsA2-4* cellulose biosynthesis gene clusters and cyclic di-GMP turnover protein genes. 11

Figure S3
Size distribution of BcsA bearing plasmids versus all plasmids of *E. coli, Shigella* and *Escherichia* spp. 13

Figure S4
Phylogenetic context of cellulose biosynthesis proteins from *E. coli.* 14

Figure S5A-E
Domain structure and length of GGDEF and EAL domain proteins encoded by BcsA encoding *E. coli* plasmids. 16

Figure S6A-C
Alignment of cyclic di-GMP turnover proteins associated with novel cellulose biosynthesis gene clusters. 22

Figure S7A,B
Phylogenetic relationship and arrangement of paralogous and xenologous BcsA cellulose synthases compared to core genome cellulose synthases encoded by individual isolates of a species. 25

Figure S8A,B
Characterisation of cellulose biosynthesis in *E. coli* 730V1 Nal^{r}. 28

Figure S9A,B,C
Comparison of cellulose production and biofilm phenotypes between *E. coli* 730V1 and 730-2 isolated from the same meat sample. 29

Figure S10
Genomic context of mobile element encoded poly-N-acetyl-glucosamine *pgaABCD* biosynthesis gene clusters. 30

Tables

Table S1
List of reference plasmids bearing alternative BcsA cellulose synthases.

Table S2
Cyclic di-GMP turnover proteins found on BcsA bearing plasmids.

Table S3
Presence of *bcsA1* and *bcsA4* cellulose synthase genes in selected meat-derived *E. coli* strains. 31

Table S4
Summary of biofilm phenotypes for *E. coli* 730V1. 32

Table S5
List of *pgaABCD* encoding mobile plasmidic elements in *E. coli.*

References 33

Supplementary Materials and Methods

Bacterial strains derived from ground beef samples and growth conditions. MacConkey agar collected generic *E. coli* isolates (n=11) from 9 US ground beef samples archived at US Meat Animal Research Center including Shiga-toxin producing *E. coli* (*stx*$^+$ *eae*$^-$, STEC, 1 strain), enterohaemorrhagic EHEC *E. coli* (*stx*$^+$ *eae*$^+$, EHEC, 1), enteropathogenic EPEC *E. coli* (*stx*$^-$ *eae*$^+$, EPEC, 1) were used to screen for core genome *bcs* EcType 1 and *bcs* EcType 4 cellulose biosynthesis operon genes. Comparison of E. coli 730V1 and 730-2 was performed in LB-NS (no salt), LB-NS diluted 1:2 fold with water and M9 minimal medium with 0.2% glucose as stated.

In silico and PCR Screening for the *bcs* EcType 4 operon. Two singleplex PCR reactions were designed to screen for novel *bcsA4* and *bcsB4* genes. Primers (bcsA_F_MG2, TCCGAGCTATAACGAACCGC/bcsA_R_MG2, GCGGAAAATCTGCGTCATCC) and *bcsB* (bcsB_F_MG2, TCCCGGTTGGCAAAGCATAT/ bcsB_R_MG2, AGGAGAGATTGGGCAACAGC). Primers were designed to target unique regions of novel plasmid born *bcsA4* of strain 730-V1. Additionally, the isolates were screened for presence of classic *bcsA1* using primers (bcsA_F_MG3, ACTGATCTTCCTGACTGCGC/bcsA_R_MG4, AACCACCGTCGGAGAAATCC) derived from conserved regions of *bcsA1*. Product was obtained after 35 cycles with an annealing temperature of 56 °C and 30 s extension time. PCR products were detected after electrophoresis in 1.5% agarose gel stained with gel red (Phenix Research Products, Berkeley, CA).

Evaluation of Congo red binding phenotype. To comparatively examine Congo red binding, *E. coli* strains 730V1 and 730-2 were inoculated on T-medium agar plates (Collinson, Emody et al., 1991) containing 1% Tryptone, 18 mg/L Congo red, 12 mg/L Coomassie brilliant blue R-250 and 1.5% agar. Inoculated plates were incubated for 24 h at 37 °C or 42 °C, ambient atmospheric oxygen or reduced oxygen conditions. Incubated plates were maintained for 3-4 h at room temperature to allow full color development. Colonies were visually inspected and colony morphotype development compared to the positive control strain 43894 OR22/30 (Uhlich, Keen et al., 2001).

Biofilm formation on microtiter plate. To assess biofilm formation in polystyrene 96-well microtiter plates, 100 µL cell suspension of OD_{600}=0.0045 was placed in wells. The plate was incubated at 42 °C in a humidity chamber without shaking, under ambient atmospheric and reduced oxygen conditions. After 72 and 96 h incubation, supernatant was removed, attached biofilm was washed with water and stained with 0.1% crystal violet for 45 min. Unbound CV was washed with water then bound CV was extracted with absolute ethanol. Biofilm mass was estimated by absorbance at 595 nm.

Cell aggregation in broth culture. To evaluate biofilm aggregates *E. coli* 730V1 and 730-2 were grown in LB-NS and M9 minimal medium. Cell suspensions with starting OD_{600}=0.0045 were incubated either under aerobic or two different microaerophilic conditions. Twentyfive mL of cell suspension in 125 mL volume flask was incubated with shaking at 200 rpm for 48 h at 42 °C, under atmospheric oxygen or with 5% O_2/10%CO_2 atmosphere created by gas pak. Microaerophilic conditions were also created by reducing headspace due to higher media to flask ratio (75 mL of media in 125 mL flask) incubated with shaking at 150 rpm for 48 h at 42 °C. Cultures were visually inspected, then transferred to 50 mL conical tubes and mixed well before recording the OD_{600} (T0). The culture tubes were left undisturbed for 1 h at room temperature, at the end of which, OD_{600} of supernatant was measured (Tn). The difference in OD and the percentage of change in OD_{600} between T0 and Tn was calculated.

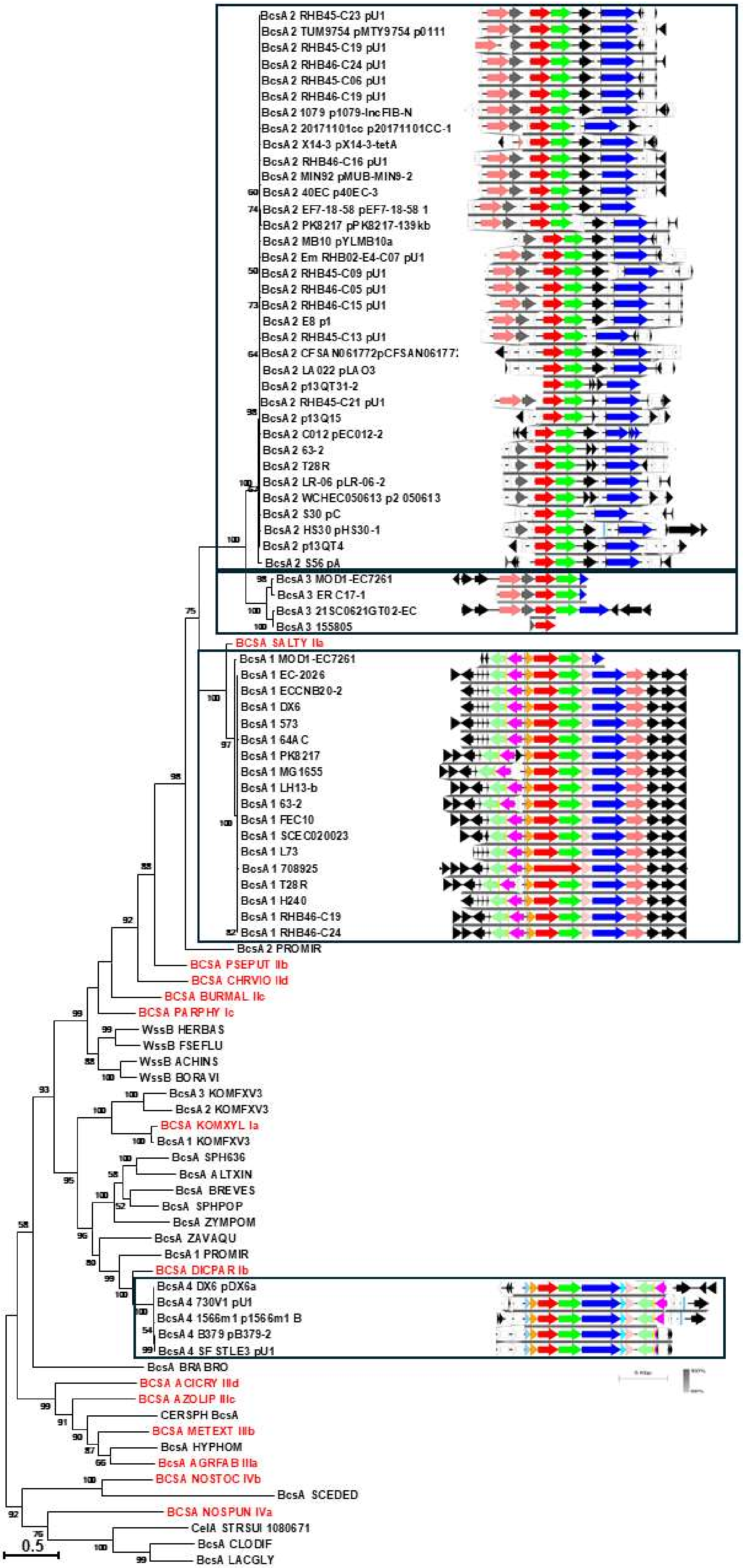
BcsA 2 RHB45-C23 pU1
BcsA 2 TUM9754 pMTY9754 p0111
BcsA 2 RHB45-C19 pU1
BcsA 2 RHB46-C24 pU1
BcsA 2 RHB45-C06 pU1
BcsA 2 RHB46-C19 pU1
BcsA 2 1079 p1079-IncFIB-N
BcsA 2 20171101cc p20171101CC-1
BcsA 2 X14-3 pX14-3-tetA
BcsA 2 RHB46-C16 pU1
BcsA 2 MIN92 pMUB-MIN9-2
BcsA 2 40EC p40EC-3
BcsA 2 EF7-18-58 pEF7-18-58 1
BcsA 2 PK8217 pPK8217-139kb
BcsA 2 MB10 pYLMB10a
BcsA 2 Em RHB02-E4-C07 pU1
BcsA 2 RHB45-C09 pU1
BcsA 2 RHB46-C05 pU1
BcsA 2 RHB46-C15 pU1
BcsA 2 E8 p1
BcsA 2 RHB45-C13 pU1
BcsA 2 CFSAN061772pCFSAN06177
BcsA 2 LA022 pLAO3
BcsA 2 p13QT31-2
BcsA 2 RHB45-C21 pU1
BcsA 2 p13Q15
BcsA 2 C012 pEC012-2
BcsA 2 63-2
BcsA 2 T28R
BcsA 2 LR-06 pLR-06-2
BcsA 2 WCHEC050613 p2 050613
BcsA 2 S30 pC
BcsA 2 HS30 pHS30-1
BcsA 2 p13QT4
BcsA 2 S56 pA
BcsA 3 MOD1-EC7261
BcsA 3 ER C17-1
BcsA 3 21SC0621GT02-EC
BcsA 3 155805
BCSA SALTY IIa
BcsA 1 MOD1-EC7261
BcsA 1 EC-2026
BcsA 1 ECCNB20-2
BcsA 1 DX6
BcsA 1 573
BcsA 1 64AC
BcsA 1 PK8217
BcsA 1 MG1655
BcsA 1 LH13-b
BcsA 1 63-2
BcsA 1 FEC10
BcsA 1 SCEC020023
BcsA 1 L73
BcsA 1 708925
BcsA 1 T28R
BcsA 1 H240
BcsA 1 RHB46-C19
BcsA 1 RHB46-C24
BcsA 2 PROMIR
BCSA PSEPUT IIb
BCSA CHRVIO IId
BCSA BURMAL IIc
BCSA PARPHY Ic
WssB HERBAS
WssB FSEFLU
WssB ACHINS
WssB BORAVI
BcsA 3 KOMFXV3
BcsA 2 KOMFXV3
BCSA KOMXYL Ia
BcsA 1 KOMFXV3
BcsA SPH636
BcsA ALTXIN
BcsA BREVES
BcsA SPHPOP
BcsA ZYMPOM
BcsA ZAVAQU
BcsA 1 PROMIR
BCSA DICPAR Ib
BcsA 4 DX6 pDX6a
BcsA 4 730V1 pU1
BcsA 4 1566m1 p1566m1 B
BcsA 4 B379 pB379-2
BcsA 4 SF STLE3 pU1
BcsA BRABRO
BCSA ACICRY IIId
BCSA AZOLIP IIIc
CERSPH BcsA
BCSA METEXT IIIb
BcsA HYPHOM
BcsA AGRFAB IIIa
BCSA NOSTOC IVb
BcsA SCEDED
BCSA NOSPUN IVa
CelA STRSUI 1080671
BcsA CLODIF
BcsA LACGLY
0.5

Figure S1. *E. coli* encoded cellulose synthases in the phylogenetic context.

S1A. Extended phylogenetic tree of BcsA proteins from *E. coli* and BcsA reference proteins including the genomic context. BcsA cellulose synthases (Table S1) were aligned with Clustal Omega and subsequently manually curated. The phylogenetic relationship has been assessed using a maximum likelihood (ML) based phylogenetic tree with 1000 ultrafast bootstraps with IQ-TREE 2. The nucleotide sequences and annotations of the *bcs* gene clusters and their gene neighborhoods were extracted from the genomic sequences stored at NCBI and aligned and visualized with Easyfig (Sullivan, Petty et al., 2011). Gene annotation, see Figure 1. Size bar, substitutions per site.

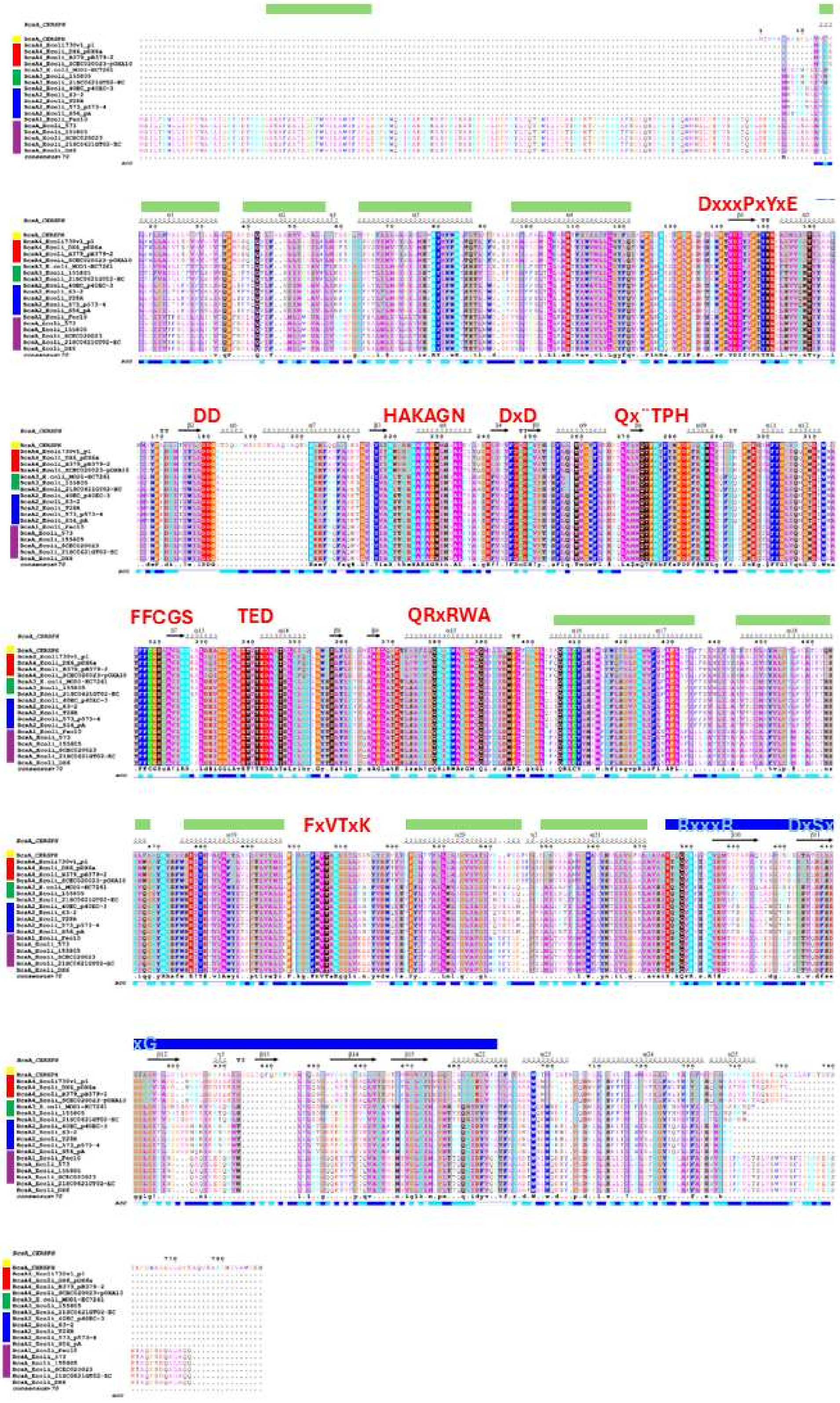

S1B. Alignment of representative BcsA2-4 cellulose synthases from *E. coli* with reference proteins including BcsA1 cellulose syntheases of *E. coli.* Conserved motifs and identified transmembrane (TM) helices characteristic for type 2 glycosyltransferases including cellulose synthases are indicated.

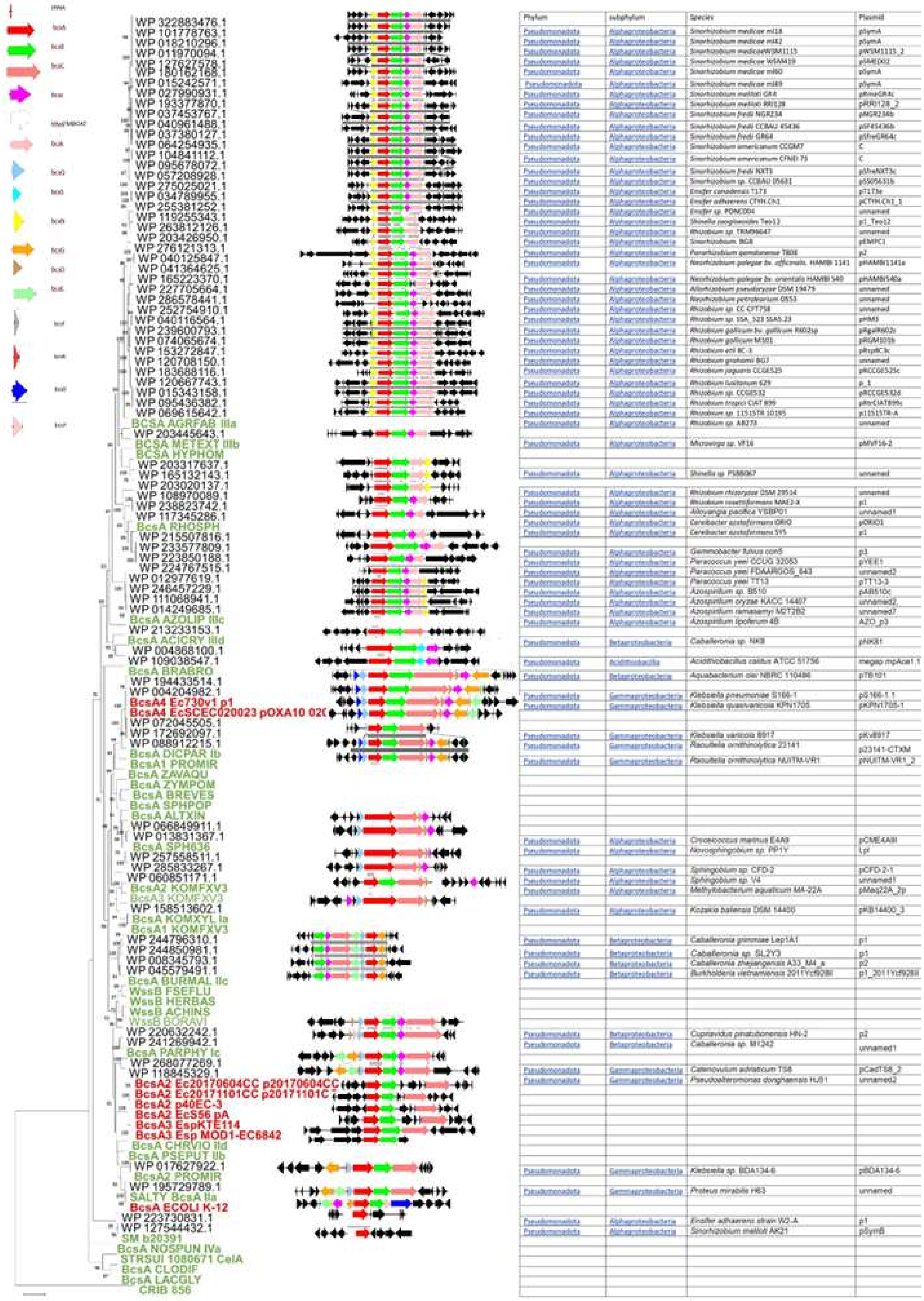

S1C. Phylogenetic tree of BcsA proteins of non-*E. coli* plasmids with BcsA protein from *E. coli* and BcsA reference proteins including their genomic context. BcsA cellulose synthases (see Figure S1A and Table S1 for reference proteins) were aligned with ClustalX 2.1 and subsequently manually curated. The phylogenetic relationship has been assessed using a maximum likelihood (ML) based phylogenetic tree with 1000 bootstraps in MEGA 11.0. The nucleotide sequences and annotations of the *bcs* gene clusters and their gene neighborhoods were extracted from the genomic sequences stored at NCBI and aligned and visualized with Easyfig (Sullivan et al., 2011).

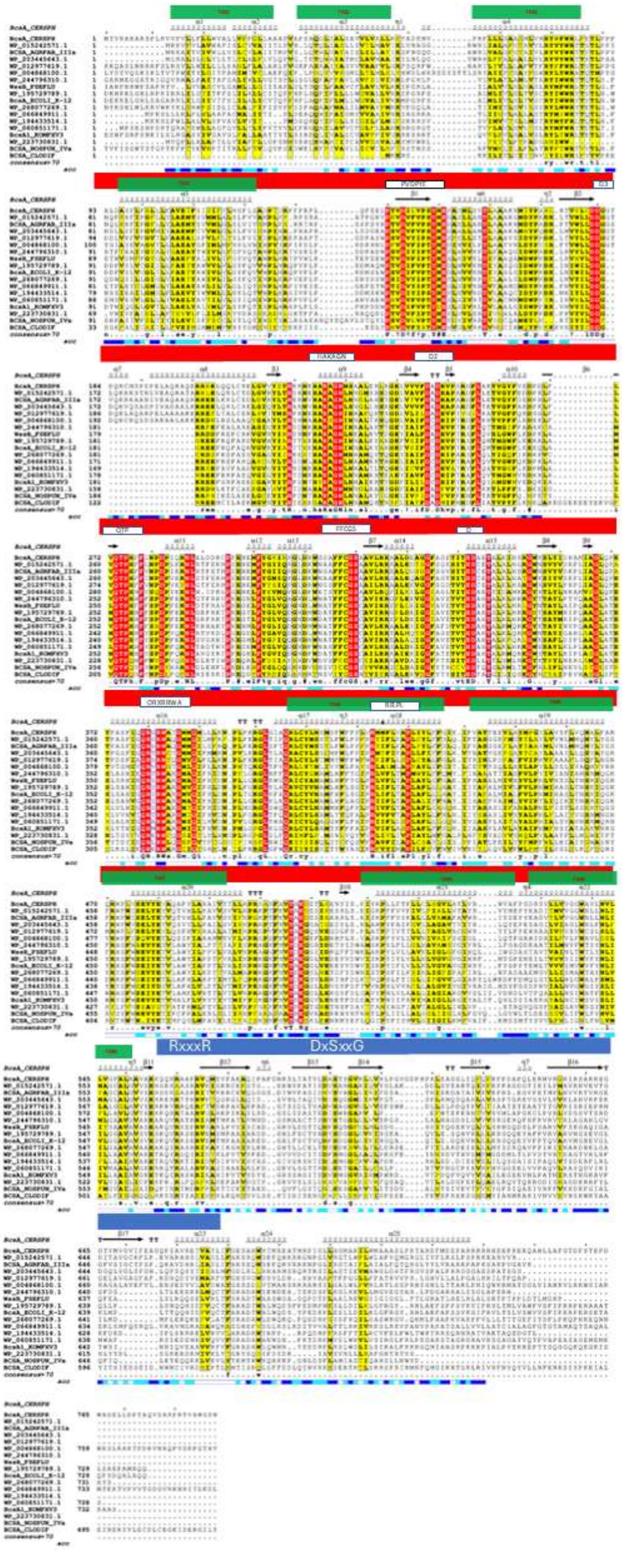

S1D. Alignment of cellulose synthases from non-*E. coli* plasmids with reference BcsA proteins. Conserved motifs and identified transmembrane (TM) helices characteristic for glycosyltransferases including cellulose synthases are indicated.

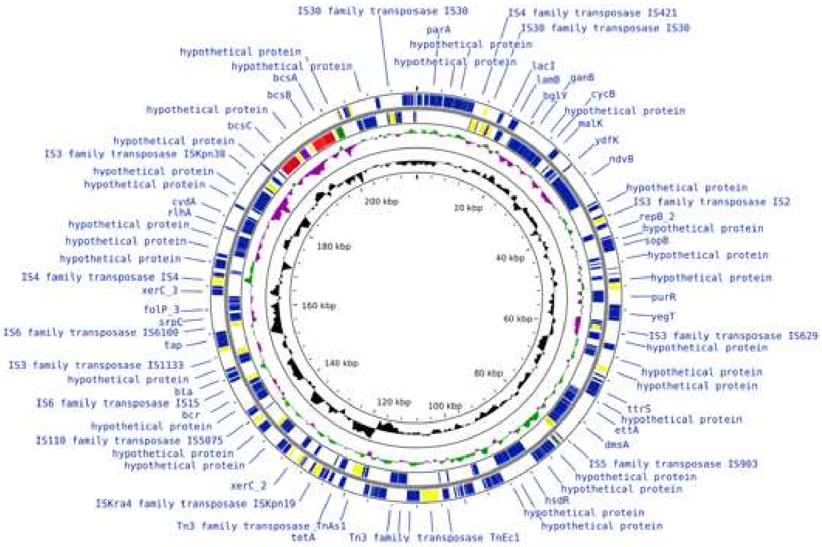

BcsA2 *E. coli* p201780604CC-1

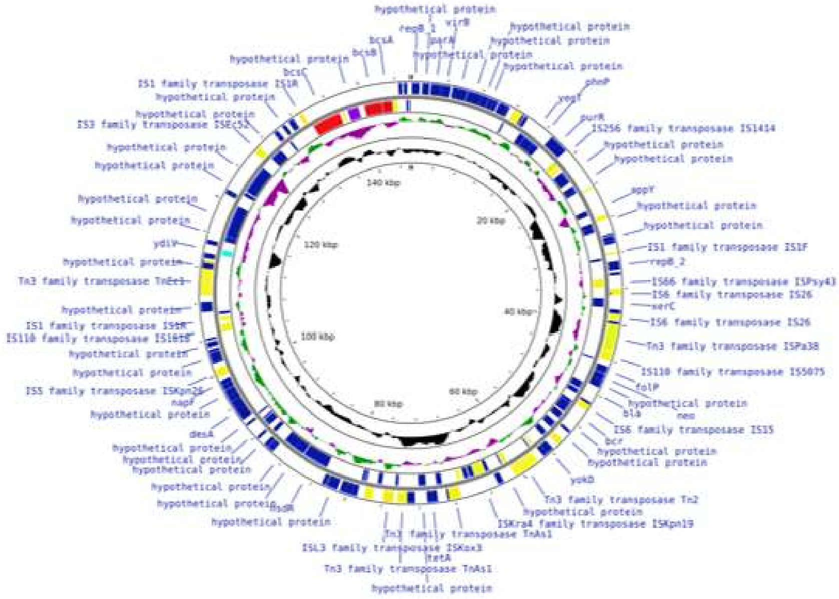

BcsA3 *E. coli* L37-2 pL37-2

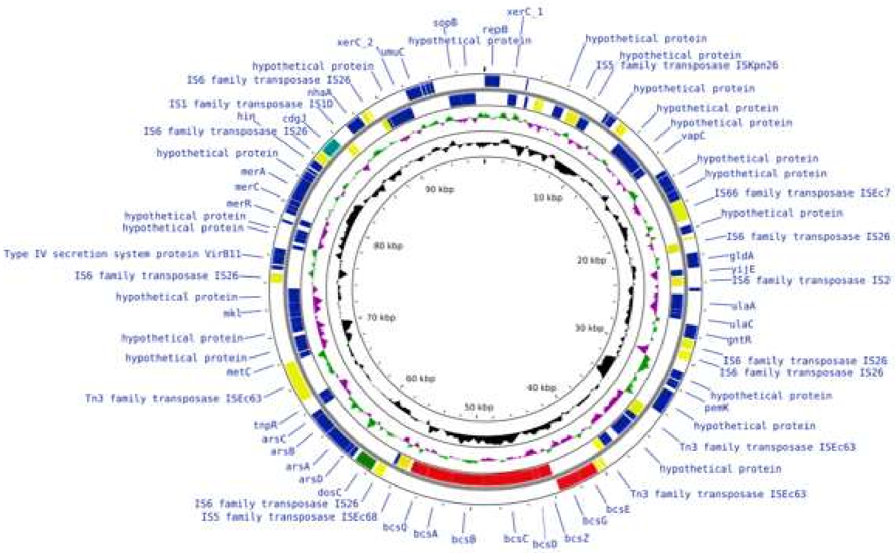

BcsA4 *E. coli* 730V1 pU1

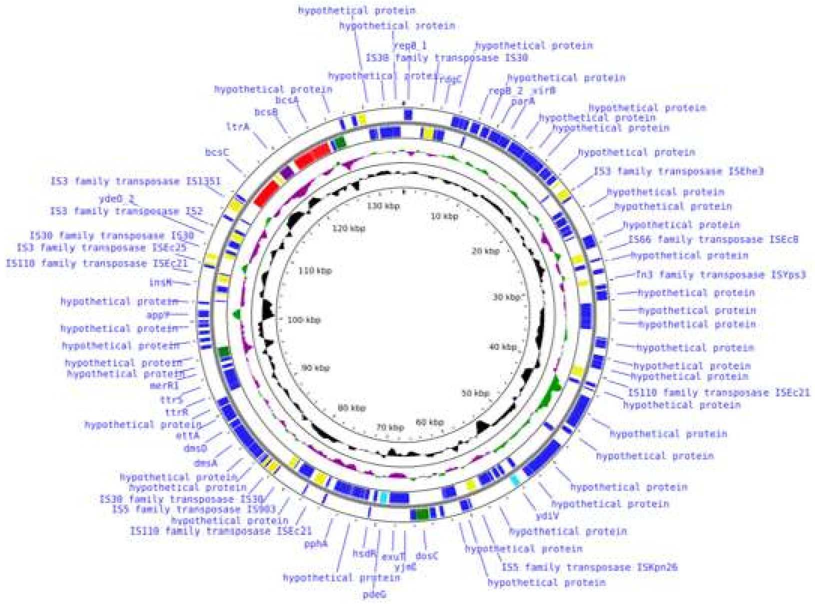

BcsA2 *E. coli* MB10 pYLMB10a

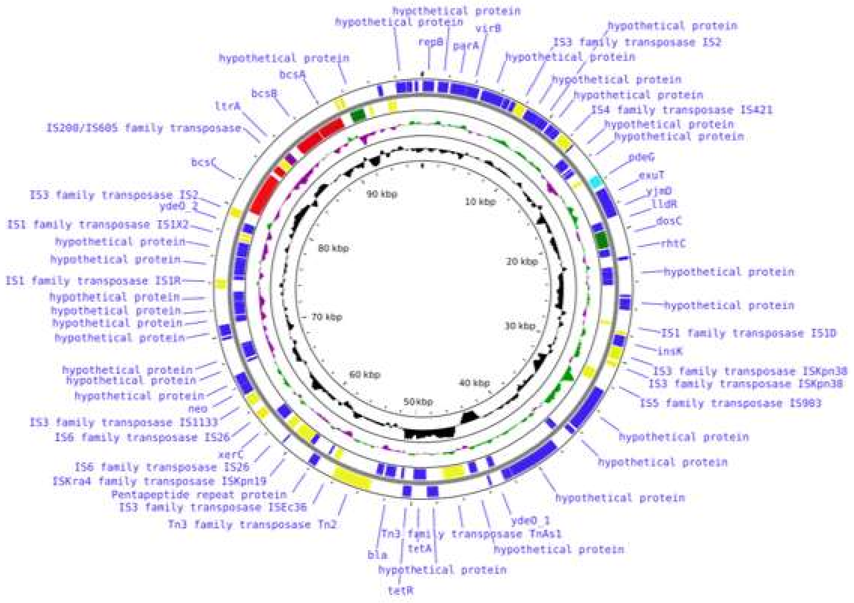

***E. coli* CUVM53 pCUVM53-2**

Figure S2. Representative plasmids harboring the different EcTypes *bcsA2-4* cellulose biosynthesis gene clusters and cyclic di-GMP turnover protein genes.

Representative plasmids of the different classes harbouring *bcsA2-4* cellulose biosynthesis gene clusters and plasmids with numerous GGDEF/EAL domain proteins (*E. coli* MB10 pYLMB10a and *E. coli* CUVM53 pCUVM53-2). In red, cellulose biosynthesis genes; green, GGDEF diguanylate cyclases; cyan, EAL phosphodisterases; yellow, transposases. Plasmids were visualized with Proksee.

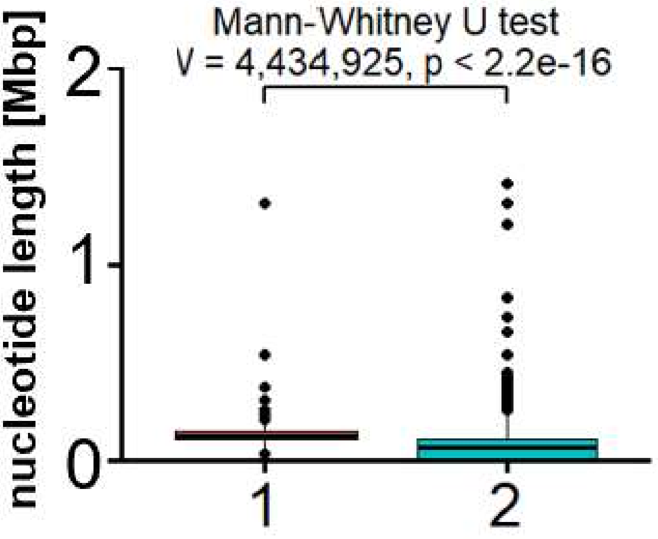

Figure S3. Size distribution of *bcsA* bearing plasmids versus all plasmids of *E. coli*, *Shigella* and *Escherichia* spp.
Size distribution of *bcsA* bearing plasmids (1) versus all plasmids of *E. coli*, *Shigella* and *Escherichia* spp. (2) as calculated with R studio version 4.4.

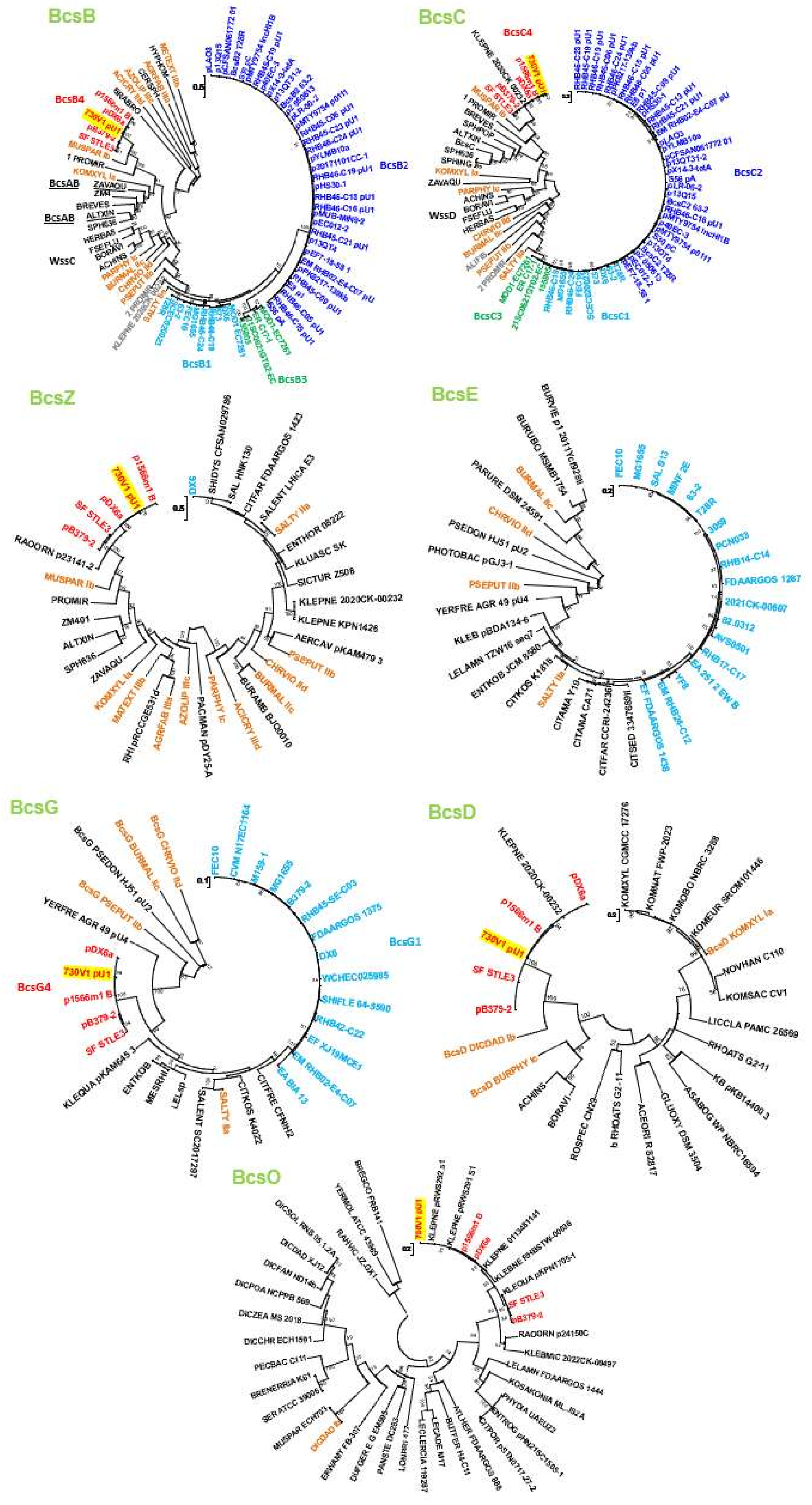
BcsB
BcsB4
BcsAB
BcsAB
WssC
BcsB1
BcsB2
BcsB3
BcsC
BcsC4
WssD
BcsC1
BcsC2
BcsC3
BcsZ
BcsE
BcsG
BcsG1
BcsG4
BcsD
BcsO

Figure S4. Phylogenetic context of cellulose biosynthesis proteins from *E. coli.*

Phylogenetic trees of BcsB, BcsC, BcsE, BcsG, BcsZ, BcsD and BcsO proteins of cellulose biosynthesis operons from BcsA encoding *E. coli* plasmids and in the phylogenetic context. Proteins were retrieved from the respective BcsA2-4 cellulose biosynthesis gene clusters and representative proteins obtained by BLAST homology search. Proteins were aligned with ClustalX 2.1 and subsequently manually curated using GeneDoc. The phylogenetic relationship has been assessed using a maximum likelihood (ML) based phylogenetic tree with 1000 bootstraps calculated and created in MEGA 11.0. Light blue designation, protein from *E. coli* EcType 1 *bcs* gene cluster; dark blue, protein from *E. coli* EcType 2 *bcs* gene cluster; green, protein from *E. coli* EcType 3 *bcs* gene cluster red, protein from *E. coli* EcType 4 *bcs* gene cluster; brown, protein from reference *bcs* gene cluster. Size bar, substitution per site.

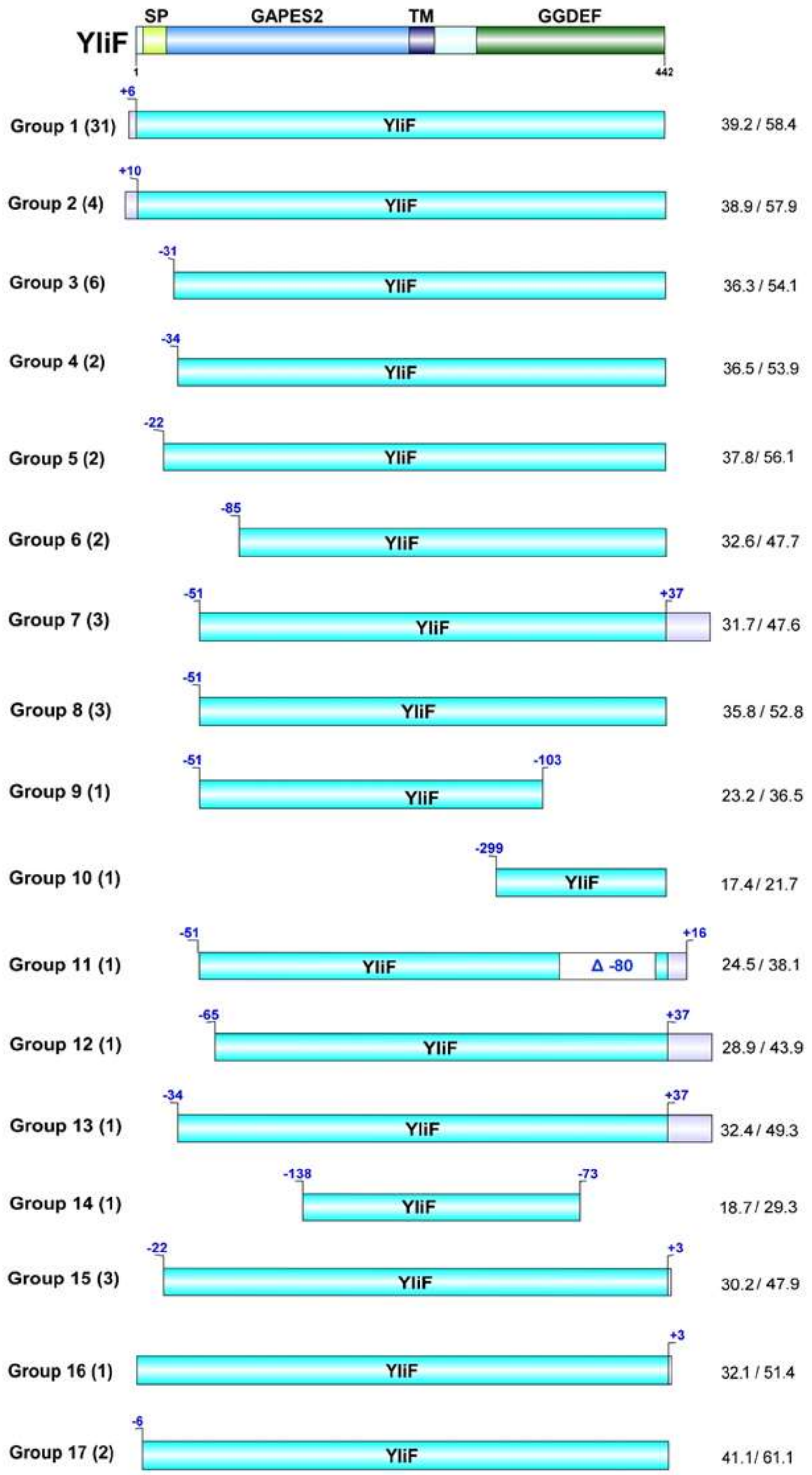

Figure S5. Domain structure and length of GGDEF and EAL domain proteins encoded by BcsA encoding *E. coli* plasmids.

S5A. YliF GAPES2-GGDEF domain protein structure compared to homologous proteins encoded on BcsA *E. coli* plasmids. On the left, the number of YliF homologous found on plasmids are indicated. On the right, the degree of identity and similarity to chromosomally encoded YliF of the reference strain *E. coli* Fec10/MG1655 is indicated.

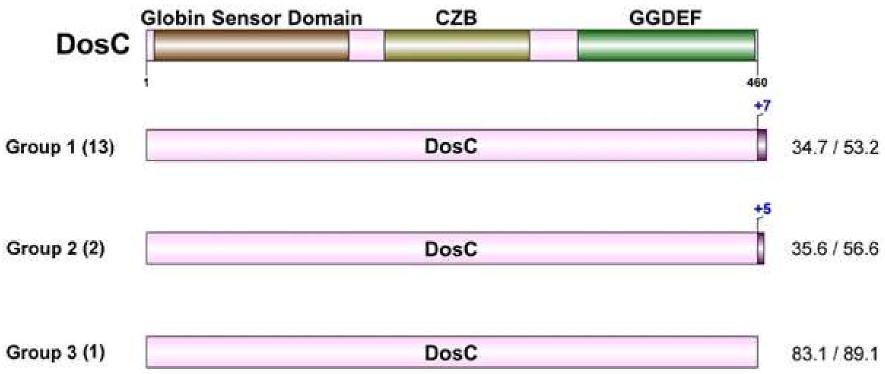

S5B. DosC Globin-Sensor-Domain-CZB-GGDEF domain protein structure compared to homologous proteins encoded on BcsA *E. coli* plasmids. On the left, the number of DosC homologous found on plasmids are indicated. On the right, the degree of identity and similarity to chromosomally encoded DosC of the reference strain *E. coli* Fec10/MG1655 is indicated.

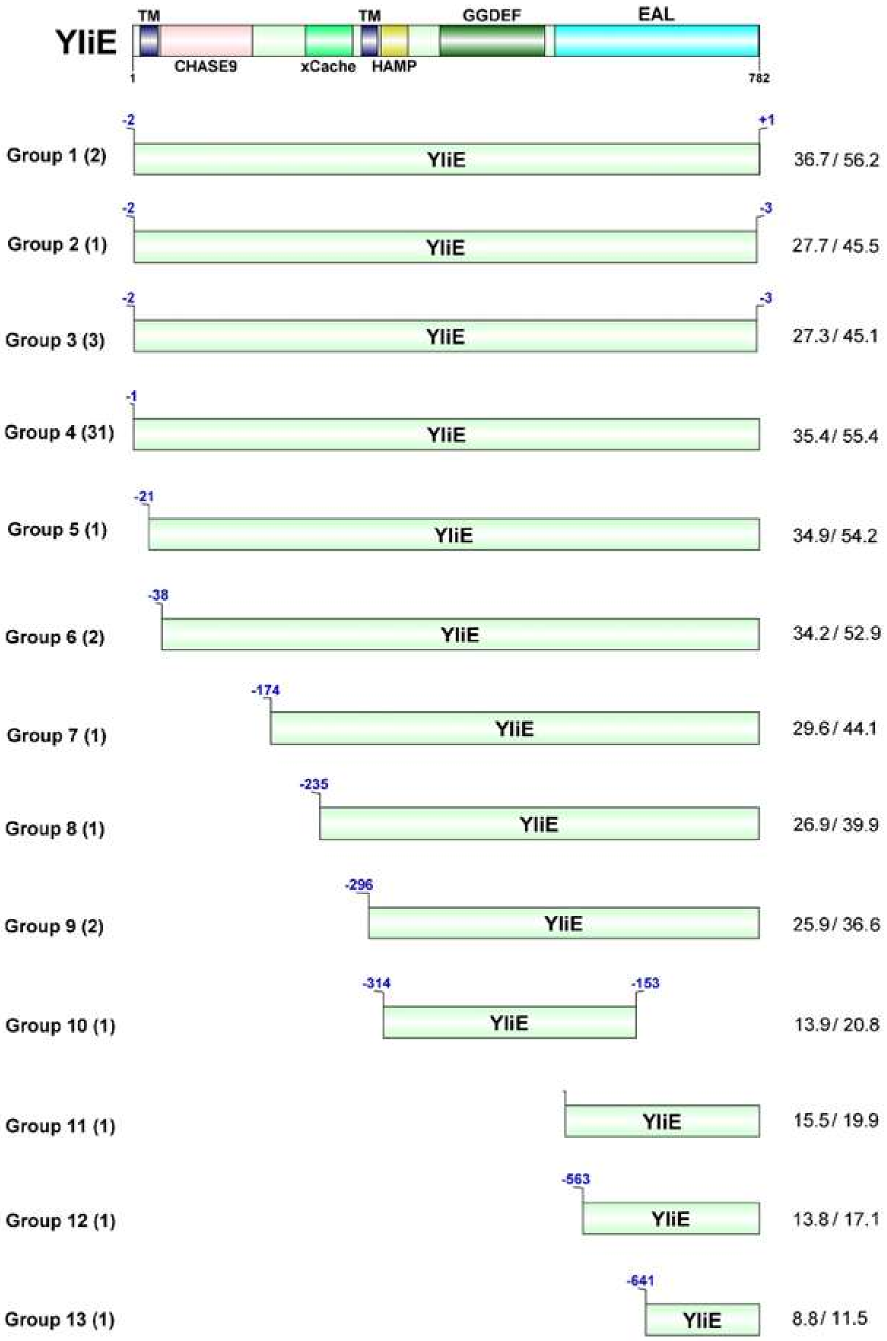

S5C. YliE TM-CHASE9-xCache-TM-HAMP-GGDEF-EAL domain protein structure compared to homologous proteins encoded on BcsA encoding *E. coli* plasmids. On the left, the number of YliE homologous found on plasmids are indicated. On the right, the degree of identity and similarity to chromosomally encoded YliE of the reference strain *E. coli* Fec10/MG1655 is indicated.

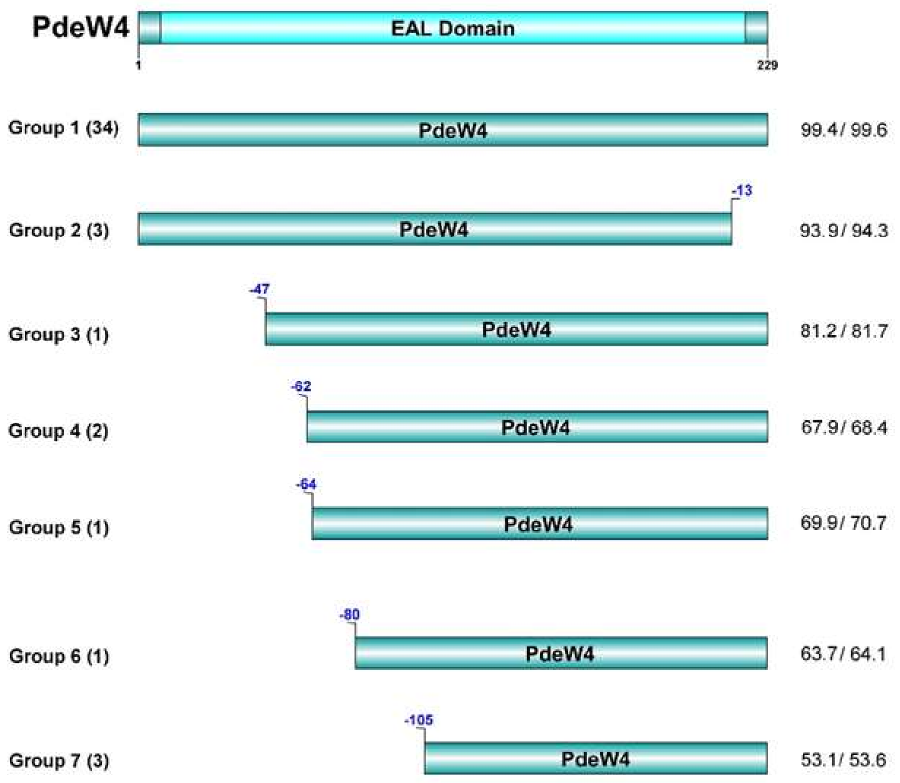

S5D. PdeW4 EAL domain protein structure compared to homologous proteins encoded on BcsA *E. coli* plasmids. On the left, the number of PdeW4 homologous found on plasmids are indicated. On the right, the degree of identity and similarity to chromosomally encoded PdeW4 is indicated.

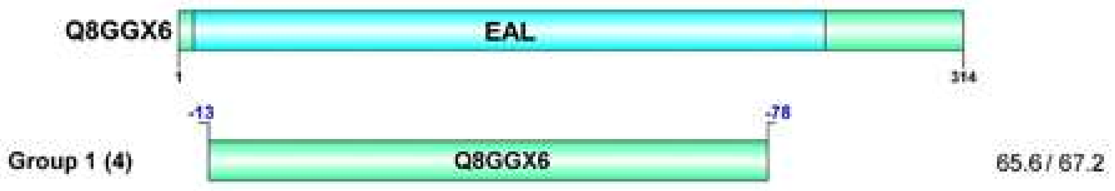

S5E. Q8GGX6 EAL domain protein structure compared to homologous proteins encoded on BcsA *E. coli* plasmids. On the left, the number of Q8GGX6 homologs found on plasmids are indicated. On the right, the degree of identity and similarity to chromosomally encoded Q8GGX6 is indicated.

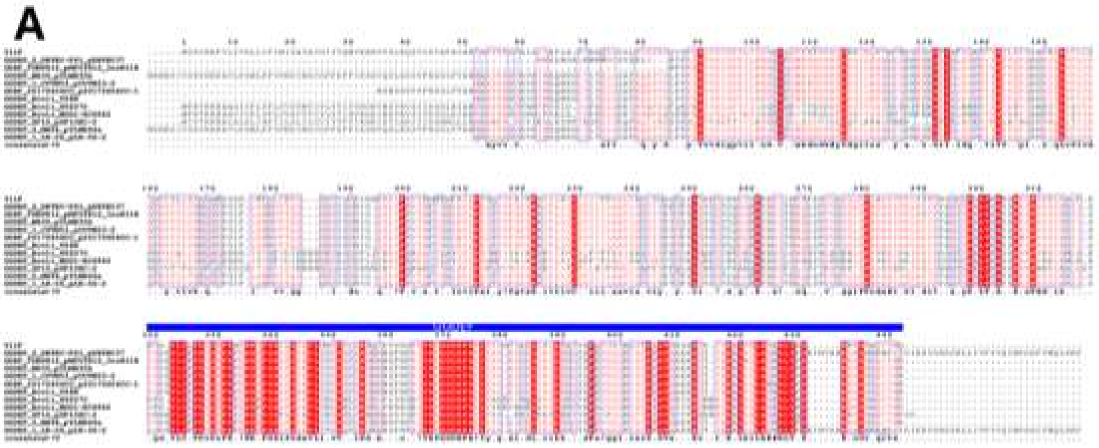

Figure S6. Alignment of cyclic di-GMP turnover proteins associated with novel cellulose biosynthesis gene clusters.

S6A. Alignment of representative YliF GGDEF domain protein encoded on BcsA bearing plasmids from *E. coli* with the chromosomally encoded YliF reference protein of the reference strain *E. coli* Fec10/MG1655. The GGDEF domain with the characteristic GGDEF motif is indicated by a blue bar.

B

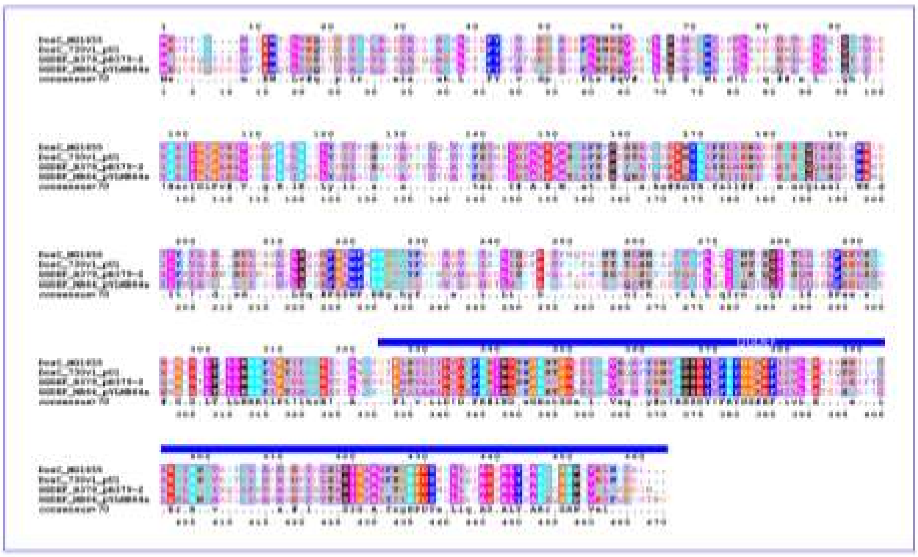

S6B. Alignment of representative DosC GGDEF domain protein encoded on BcsA plasmids from *E. coli* with the chromosomally encoded DosC reference protein of the reference strain *E. coli* Fec10/MG1655. The GGDEF domain with the characteristic GGDEF motif is indicated by a blue bar.

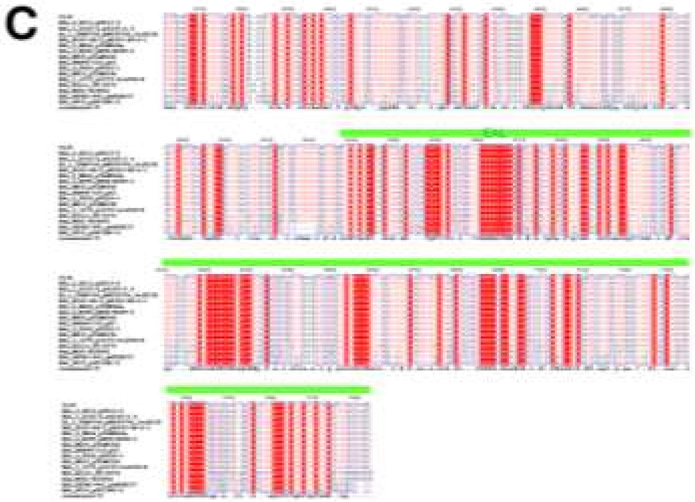

S6C. Alignment of representative YliE EAL domain protein encoded on BcsA plasmids from *E. coli* with the chromosomally encoded YliE reference protein of the reference strain *E. coli* Fec10/MG1655. The EAL domain with the characteristic EAL motif is indicated by a green bar.

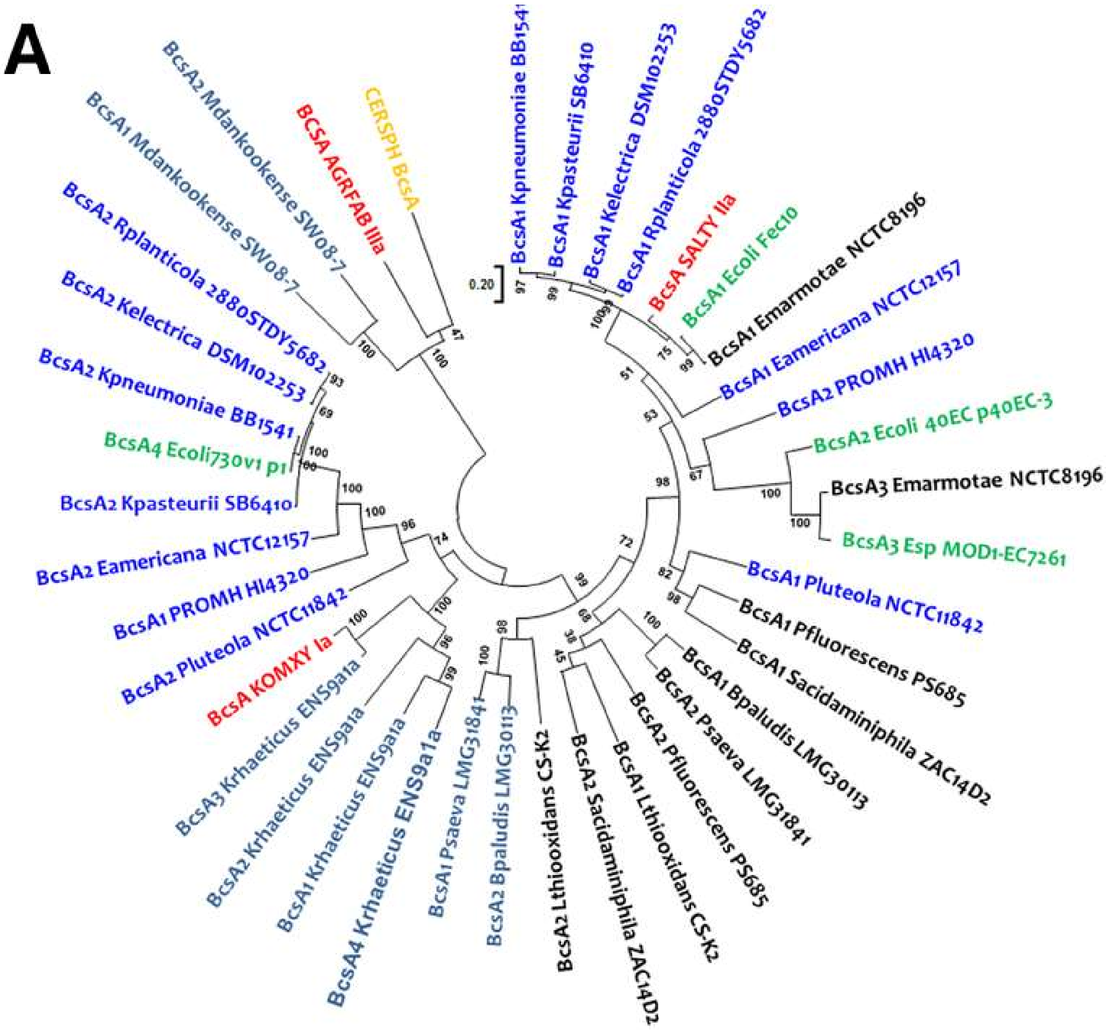

Figure S7. Phylogenetic relationship and arrangement of paralogous and xenologous BcsA cellulose synthases compared to core genome cellulose synthases encoded by individual isolates of a species.

S7A. Phylogenetic relationship of paralogous and xenologous BcsA cellulose synthases compared to core genome cellulose synthases encoded by individual isolates of a species. BcsA cellulose synthases were retrieved from Uniprot (Apweiler, Bairoch et al., 2004). BcsA cellulose synthases were aligned with ClustalX 2.1 and subsequently manually curated with GeneDoc. The phylogenetic relationship has been assessed using a maximum likelihood (ML) based phylogenetic tree with 1000 bootstraps in MEGA 7.0. Protein identities are; BcsA1_Ecoli_MG1655, P37653; BcsA1_Emarmotae_NCTC8196, WP_000025929.1; BcsA3_Emarmotae_NCTC8196, WP_000576412.1; BcsA1_Kpneumoniae_BB1541, WP_002921508.1; BcsA2_Kpneumoniae_BB1541, WP_004186066.1; BcsA1_Kpasteurii_SB6410, WP_142445849.1; BcsA2_Kpasteurii_SB6410, WP_004126352.1; BcsA1_Kelectrica_DSM102253, WP_141963125.1; BcsA2_Kelectrica_DSM102253, WP_141963111.1; BcsA1_Rplanticola_-2880STDY5682; WP_064385327.1; BcsA2_Rplanticola_2880STDY5682, WP_032690497.1; BcsA1_Eamericana_NCTC12157, WP_034790467.1; BcsA2_Eamericana_NCTC12157, WP_034794142.1; BcsA1_Pfluorescens_PS685, WP_150628339.1; BcsA2_Pfluorescens_PS685, WP_150628066.1; BcsA1_Pluteola_NCTC11842, WP_010798019.1; BcsA2_Pluteola_NCTC11842, WP_010795072.1; BcsA1_Sacidaminiphila_ZAC14D2, A0A0S1B309_9GAMM; BcsA2_Sacida-miniphila_ZAC14D2, A0A0S1B2Y2_9GAMM; BcsA1_PROMH_HI4320, B4F0R1_PROMH; BcsA2_PROMH_HI4320, B4F1A9_PROMH; BcsA1_Mdankookense_SW08-7, WP_373321913.1; BcsA2_Mdankookense_SW08-7, VUF12718.1; BcsA1_Bpaludis_LMG30113, WP_031403114.1; BcsA2_Bpaludis_LMG30113, WP_034198152.1;

BcsA1_Psaeva_LMG31841, CAG4896730.1; BcsA2_Psaeva_LMG31841, CAG4888940.1; BcsA1_Lthiooxidans_CS-K2, WP_338284421.1; BcsA2_Lthiooxidans_CS-K2, WP_130557523.1; BcsA1_Krhaeticus_ENS9a1a, QIP35774.1; BcsA2_K-rhaeticus_ENS9a1a, QIP35696.1; BcsA3_Krhaeticus_ENS9a1a, QIP34621.1; BcsA4_Krhaeticus_-ENS9a1a, QIP34889.1; BcsA3_E_coli_MOD1-EC7261, WP_309491259.1; BcsA2_Ecoli_40EC p40EC-3, WP_052248696.1; BcsA4_Ecoli 730V1 p1, WP_004186066.1; CERSPH_BcsA,Q3J125_RHOS4; BcsA_KOMXY_Ia, AHI24410.1; BcsA_SALTY_IIa, A0A0F6B898_SALT1; BCSA_AGRFAB_IIIa, A9CEZ7_AGRFC. Scale bar indicates substitutions per site.

B

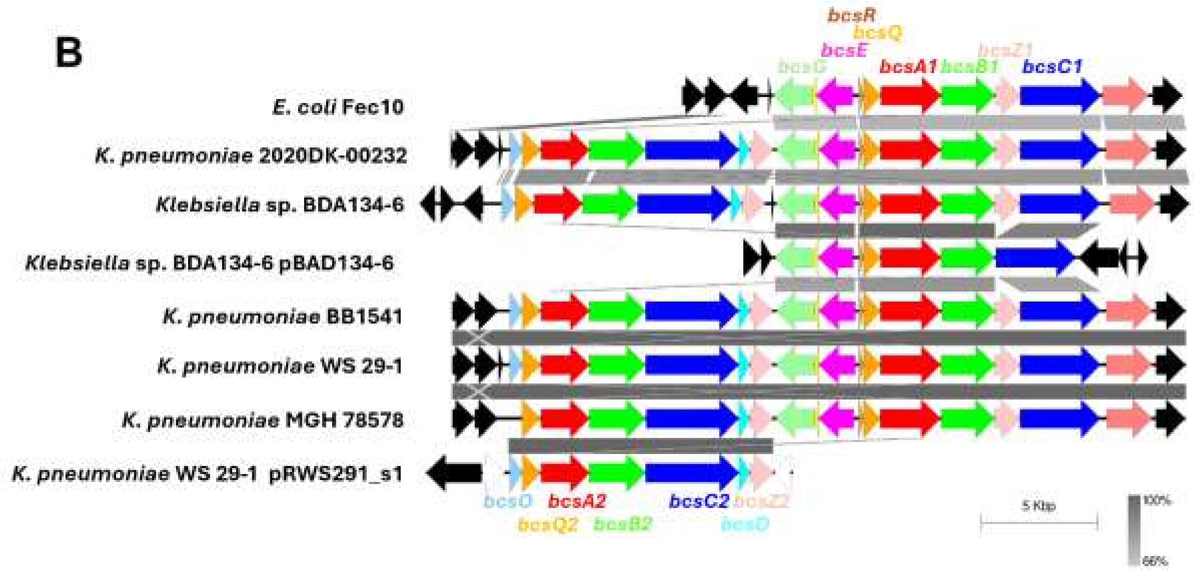

S7B. Arrangement of the two cellulose biosynthesis gene clusters in selected *K. pneumoniae* strains. BLAST analysis and visualization has been performed with Easyfig after retrieval of the genome annotations of the respective whole genome sequenced strains from NCBI. The *bcs* gene cluster from *E. coli* Fec10 is used as a reference. Located downstream of *bcsC1* is the gene encoding the GAPES3-HAMP-xGGDEF-EAL domain protein YhjK (dark skin-colored).

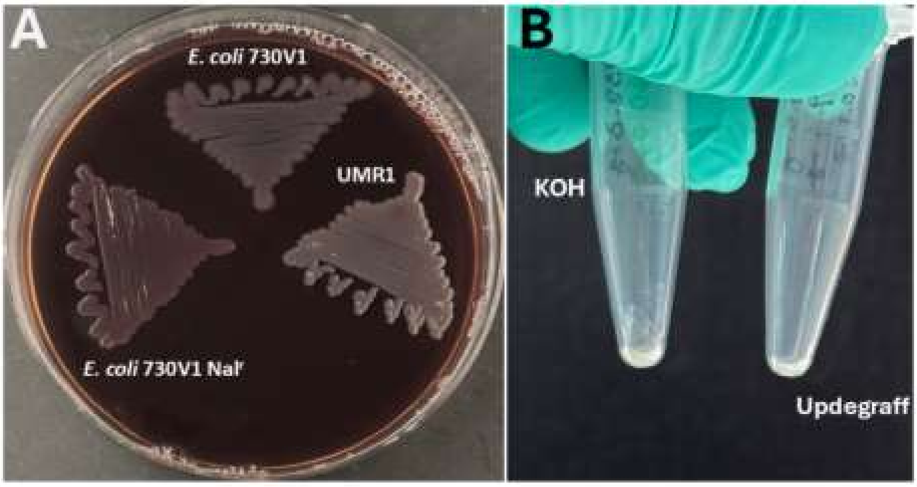

Figure S8. Characterisation of cellulose biosynthesis in *E. coli* 730V1 Nal^r.

S8A. Rdar morphotype of *E. coli* 730V1 Nal^r compared to the parent strain *E. coli* 730V1 and *S. typhimurium* UMR1. Bacterial cells were grown on a Congo red agar plate incubated for 24 h at 37 ℃ after which the plate was photographed.

S8B. Pellet of exopolysaccharide (cellulose) isolated by 1% KOH and the Updegraff approach from *E. coli* 730V1.

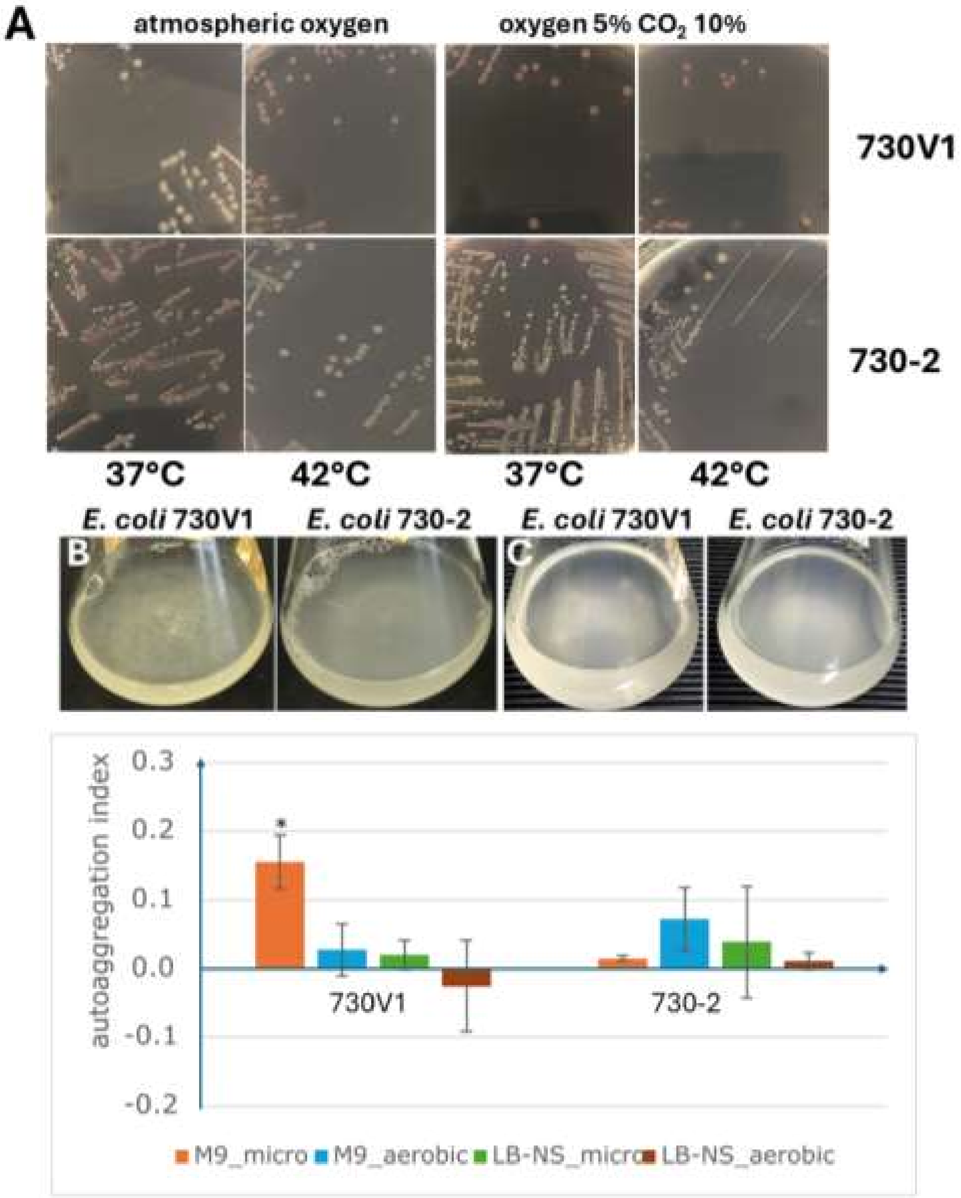

Figure S9. Comparison of cellulose production and biofilm phenotypes between *E. coli* 730V1 and 730-2 isolated from the same meat sample.

S9A. *E. coli* strain 730V1 and 730-2 were inoculated on Congo red T-medium agar plates (Collinson et al., 1991).

S9 B. and C. *E. coli* strain 730V1 and 730-2 were grown in M9 minimal medium with 0.2% glucose as carbon source under microaerophilic conditions either B. under 5% O_2/10% CO_2 atmosphere or C. by higher medium to flask volume ratio.

S9 D. Autoaggregation index displayed as absolute reduction in OD_{600} after 1.5 hours undisturbed incubation.

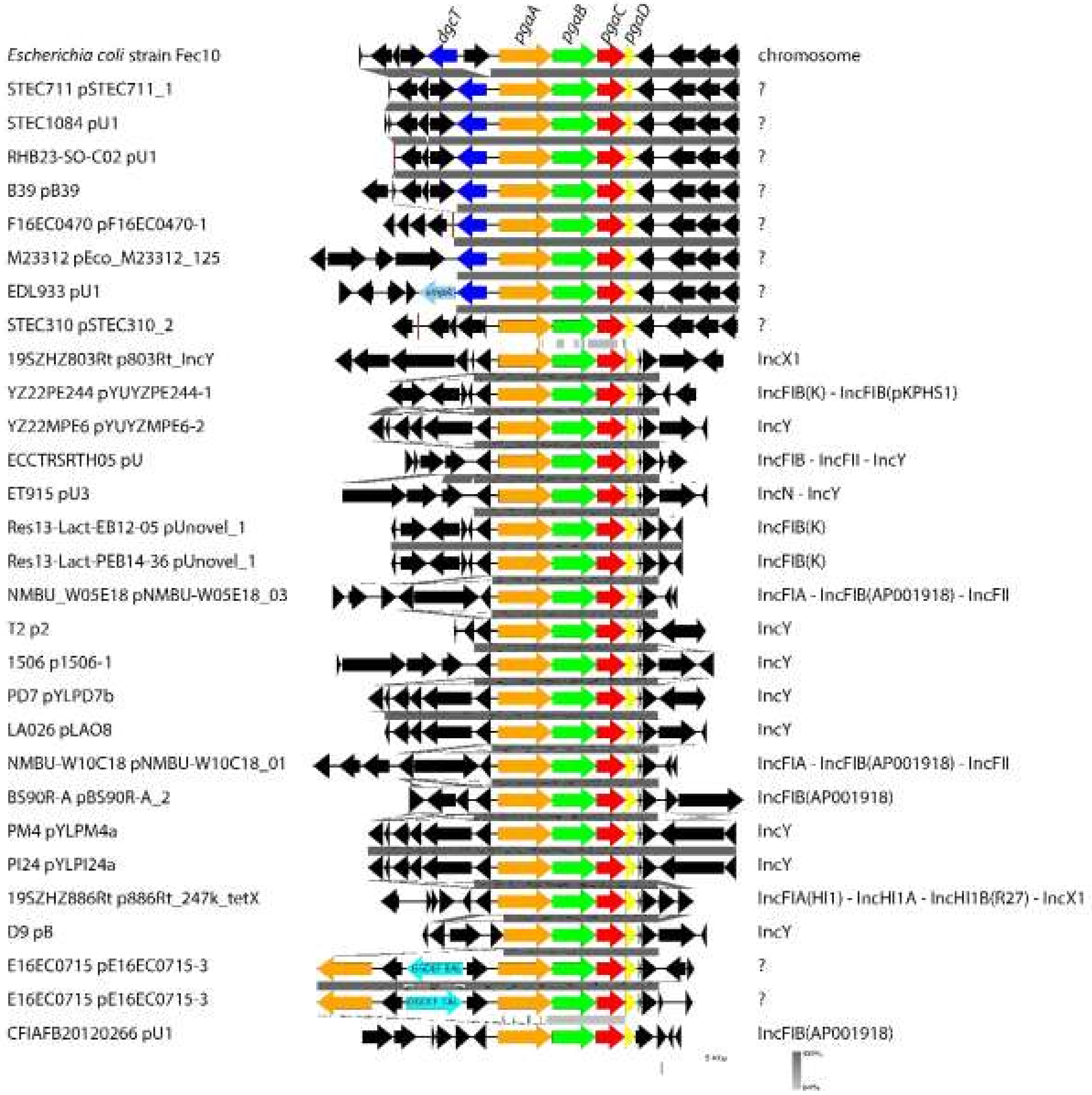

Figure S10. Genomic context of mobile element encoded poly-N-acetyl-glucosamine *pgaABCD* biosynthesis gene clusters.

Genomic context of mobile element encoded poly-N-acetyl-glucosamine *pgaABCD* biosynthesis operon in comparison with the chromosomally encoded reference *pagABCD* gene cluster. Blue arrows, cyclic di-GMP turnover genes.

Table S3. Presence of *bcsA1* and *bcsA4* cellulose synthase genes in selected meat-derived *E. coli* strains.

***E. coli* strain**	***bcsA4B4 genes***		**core genome *bcsA* gene**
	bcsA4	*bcsB4*	*bcsA1*
267-1	0	0	0
725-V2	0	0	1
728-1	0	0	1
730-2	0	0	1
730V1	1	1	0
741-1	0	0	0
745-1	0	0	1
745-V2	0	0	1
766-V2	0	0	1
792-V2	0	0	1
801-V1	0	0	1

Table S4. Summary of biofilm phenotypes for *E. coli* 730V1

37°C	42°C	M9 medium 0.2% glucose
-/-	-/-	aerobic
+/-	+/-	microaerophilic ma
+/-	+/-	ma 5% O_2 /10% CO_2

aggregation/adherence on glass, ma, microaerophilic

		**M9 medium 0.4% glucose**
-/+	n.d.	aerobic
+/+	+/+	microaerophilic

aggregation/stained with Cacoflour white

		**LB-NS medium**
-/-	-/-	aerobic
-/-	-/-	microaerophilic
-/-	-/-	ma 5% O_2/10% CO_2

aggregation/adherence on glass, ma, microaerophilic

		**96-well plate biofilm**
-	-	LB NS
-	n.d.	LB NS reduced O_2
-	-	M9 medium 0.2% glucose, 20% O_2
n.d.	-	M9 medium 0.2% glucose, reduced O_2

		**agar plate based biofilm assay**
bas	bas	LB-NS medium
saw	ras	1% Tryptone medium
ras	ras	1% Tryptone medium 5% oxygen/10% CO_2

bas, brown and smooth; saw, smooth and white; ras, red and smooth.

References

Apweiler, R., Bairoch, A., Wu, C.H., Barker, W.C., Boeckmann, B., Ferro, S. et al. (2004) Uniprot: The universal protein knowledgebase. *Nucleic Acids Res* **32**: D115-119.
Collinson, S.K., Emödy, L., Müller, K.H., Trust, T.J. & Kay, W.W. (1991) Purification and characterization of thin, aggregative fimbriae from *Salmonella enteritidis*. *J Bacteriol* **173**: 4773-4781.
Sullivan, M.J., Petty, N.K. & Beatson, S.A. (2011) Easyfig: A genome comparison visualizer. *Bioinformatics* **27**: 1009-1010.
Uhlich, G.A., Keen, J.E. & Elder, R.O. (2001) Mutations in the *csgD* promoter associated with variations in curli expression in certain strains of *Escherichia coli* O157:H7. *Appl Environ Microbiol* **67**: 2367-2370.
